# Acute Malaria Induces PD1^+^CTLA4^+^ Effector T Cells with Cell-Extrinsic Suppressor Function

**DOI:** 10.1371/journal.ppat.1005909

**Published:** 2016-11-01

**Authors:** Maria Sophia Mackroth, Annemieke Abel, Christiane Steeg, Julian Schulze zur Wiesch, Thomas Jacobs

**Affiliations:** 1 Department of Medicine I, University Medical Centre Hamburg-Eppendorf, Hamburg, Germany; 2 Department of Immunology, Bernhard-Nocht-Institute of Tropical Medicine, Hamburg, Germany; Queensland Institute of Medical Research, AUSTRALIA

## Abstract

In acute *Plasmodium falciparum* (*P*. *falciparum*) malaria, the pro- and anti-inflammatory immune pathways must be delicately balanced so that the parasitemia is controlled without inducing immunopathology. An important mechanism to fine-tune T cell responses in the periphery is the induction of coinhibitory receptors such as CTLA4 and PD1. However, their role in acute infections such as *P*. *falciparum* malaria remains poorly understood. To test whether coinhibitory receptors modulate CD4^+^ T cell functions in malaria, blood samples were obtained from patients with acute *P*. *falciparum* malaria treated in Germany. Flow cytometric analysis showed a more frequent expression of CTLA4 and PD1 on CD4^+^ T cells of malaria patients than of healthy control subjects. *In vitro* stimulation with *P*. *falciparum*-infected red blood cells revealed a distinct population of PD1^+^CTLA4^+^CD4^+^ T cells that simultaneously produced IFNγ and IL10. This antigen-specific cytokine production was enhanced by blocking PD1/PDL1 and CTLA4. PD1^+^CTLA4^+^CD4^+^ T cells were further isolated based on surface expression of PD1 and their inhibitory function investigated *in-vitro*. Isolated PD1^+^CTLA4^+^CD4^+^ T cells suppressed the proliferation of the total CD4^+^ population in response to anti-CD3/28 and plasmodial antigens in a cell-extrinsic manner. The response to other specific antigens was not suppressed. Thus, acute *P*. *falciparum* malaria induces *P*. *falciparum*-specific PD1^+^CTLA4^+^CD4^+^ T_effector_ cells that coproduce IFNγ and IL10, and inhibit other CD4^+^ T cells. Transient induction of regulatory T_effector_ cells may be an important mechanism that controls T cell responses and might prevent severe inflammation in patients with malaria and potentially other acute infections.

## Author Summary

In acute infections like malaria, our immune systems must achieve a careful balance between inflammatory and anti-inflammatory responses to successfully fight the infection without causing harm to the host. In this study, we examined the CD4^+^ T cell response and CD4^+^ T cell regulation in patients with acute malaria. Important mechanisms to control CD4^+^ T cell activity include specific regulatory T cell populations which suppress other T cells or the expression of so-called coinhibitory receptors which inhibit the inflammatory response of the expressing cells. We showed that in malaria patients, high numbers of T cells expressed the coinhibitory receptors CTLA4 and PD1. Surprisingly, despite the high expression of coinhibitory receptors, malaria-specific effector function was predominantly found in the PD1^+^CTLA4^+^CD4^+^ T cell population. A blockade of the receptors enhanced the effector response. Even more surprising, we found that although PD1^+^ CTLA4^+^CD4^+^ T cells contained the majority of malaria-specific T cells, they showed, at the same time, cell-extrinsic suppressor activity and actively downregulated T cell proliferation. Thus, our observations describe a new population of “regulatory” T effector cells which are induced during acute malaria. This will contribute to our understanding of the complex immune pathways activated during acute malaria.

## Introduction

Malaria remains one of the leading health burdens worldwide with about 600 000 deaths per year. Most of these deaths are attributable to the species *Plasmodium falciparum (P*. *falciparum)* [1]. Primary infection with *P*. *falciparum* initially induces a strong Th1-type CD4^+^ T cell response. While a strong proinflammatory Th1 response can contribute to control of parasitemia and protection to subsequent infections [2, 3], it can also be pathological as it activates the endothelium and thereby promotes sequestration of parasitized red blood cells in the microvessels of vital organs such as the brain [4, 5]. This impedes parasite clearance by the spleen and enhances severe manifestations of malaria such as cerebral malaria [6–8]. Therefore, a tight coordination of the immune response is needed to ensure the optimal outcome for the patient. Strong proinflammatory responses activate counteracting pathways such as the induction of regulatory T cell (T_reg_) populations and the production of anti-inflammatory cytokines, which both are crucial for preventing immunopathology in malaria and other parasitic diseases [9–12]. Another key mechanism that regulates potentially immunopathological T cell responses in the periphery is the induction of coinhibitory receptors such as cytotoxic T-Lymphocyte attenuator 4 (CTLA4) and programmed death 1 (PD1) on T cells.

The importance of PD1 and CTLA4 in T cell regulation has largely been studied in various chronic viral diseases, including HIV, hepatitis B, and hepatitis C. Such chronic viral diseases induce sustained PD1 and CTLA4 expression on activated T cells. This is associated with T cell exhaustion and reduced effector functions of the cells. Blockade of these receptors can partially rescue the T cell responses to these viruses, thereby reducing the viral burden [13–17]. Several recent studies, however, suggest that coinhibitory receptors inhibit T effector functions not only in chronic viral but also in acute infections [18–22]. In support of this, we and other groups have shown that in experimental murine malaria, conventional T cells strongly express the coinhibitory receptors CTLA4 and PD1 [18, 23]. Blocking coinhibitory receptors improves parasite control [20, 24, 25] but also leads to more severe manifestations of disease in several models of experimental malaria [18, 20, 23]. Recent studies reported that CTLA4 and PD1 are also upregulated on the T cells of patients with acute malaria or children that are regularly exposed to *P*. *falciparum* in endemic areas [24, 26–28]. However, it remains unclear how the expression of these coinhibitory receptors influences the immune response to acute *P*. *falciparum* infection in humans.

We evaluated the CD4^+^ T cell response in patients with acute imported malaria in Hamburg, Germany to investigate whether the induction of coinhibitory receptors downregulates the T cell response in acute *P*. *falciparum* malaria and to further elucidate involved regulatory pathways. High numbers of CD4^+^ T cells in the peripheral blood of malaria patients expressed CTLA4 and PD1. These PD1^+^CTLA4^+^CD4^+^ T cells showed two distinct functions. Firstly, they proliferated and produced IFNγ and IL10 in response to *P*. *falciparum*. Blockade of CTLA4 and PD1 markedly increased *P*. *falciparum*-specific cytokine production. Secondly, these T cells also suppressed the proliferation of other CD4^+^ T cells in response to polyclonal and *P*. *falciparum*-specific stimulation. Thus, PD1^+^CTLA4^+^CD4^+^ T cells may be a new population of regulatory T_effector_ (T_eff_) cells that arises from the CD4^+^ T_eff_ population during acute infection to downregulate proinflammatory and potentially immunopathological responses.

## Results

### Malaria-specific CD4^+^ T_eff_ cells are autoregulated by CTLA4 and PD1

#### Induction of PD1^+^ and CTLA4^+^CD4^+^ T cells during acute *P*. *falciparum* malaria

**Fig 1:**
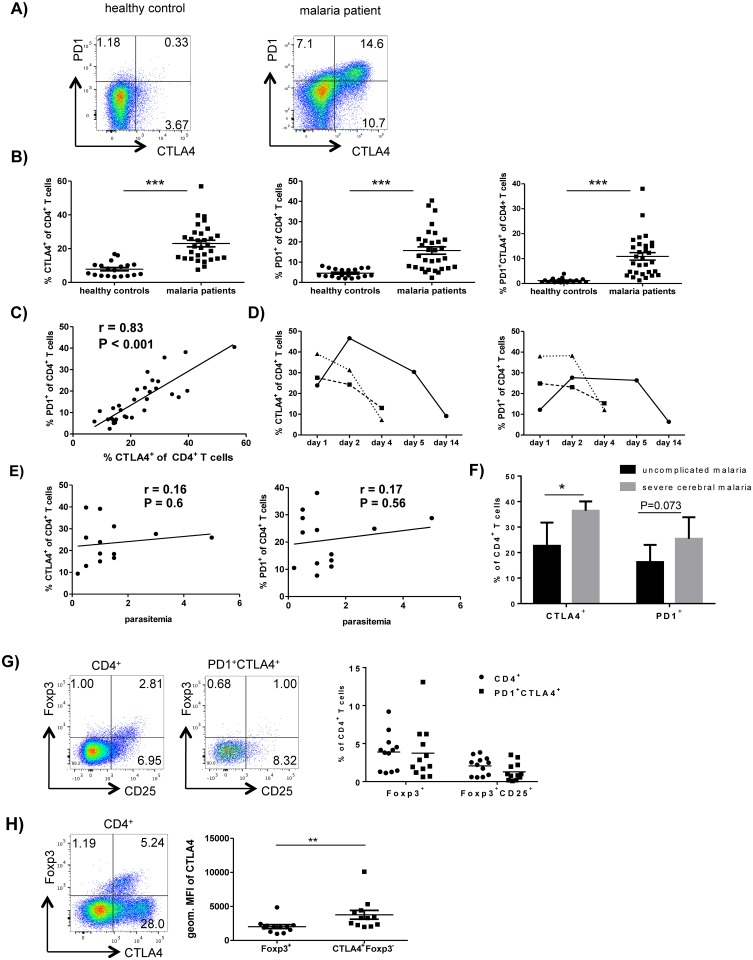
Acute *P*. *falciparum* malaria leads to a strong induction of CTLA4 and PD1 on conventional CD4^+^ T cells. A) Blood samples from acute malaria patients and healthy controls were analyzed *ex-vivo* for the expression of PD1 and intracellular CTLA4 on CD4^+^ T cells by flow cytometry. Dotplots show one representative donor out of 31 (malaria patients) or 19 (healthy controls). B) Scatter plots show the frequency of CTLA4^+^ (left), PD1^+^ (middle) and PD1^+^CTLA4^+^ cells (right) as percentage of CD4^+^ T cells for all analyzed donors. Horizontal bars represent means. ***, P < 0,0001 (t-test with Holm-Sidak correction for multiple comparisons). Gating strategies for CD4^+^ T cells and CTLA4^+^ and PD1^+^CD4^+^ T cells are presented in S3 Fig. C) Correlation analysis between the expression levels of PD1 and intracellular CTLA4 on CD4^+^ T cells for all malaria patients analyzed (N = 31). The linear regression line is shown. R = 0.83, P < 0,001 (Pearson correlation). D) Kinetics of CTLA4 (left) and PD1 (right) expression in CD4^+^ T cells at the time of diagnosis (day 1) and over the time-course of the malaria treatment and follow-up. Three malaria patients are shown. E) Relationship between *P*. *falciparum* parasitemia at time of diagnosis and expression of CTLA4 (left) and PD1 (right) on CD4^+^ T cells at the same time point. The linear regression lines are shown. R = 0.16 and R = 0.17 (Pearson correlation). F) The bar graphs shows the frequency of CTLA4^+^ and PD1^+^ cells as percentage of CD4^+^ T cells for donors with known uncomplicated malaria (n = 13) and severe, cerebral malaria (n = 3). *, P = 0.024 and NS, P = 0.073 (unpaired t-test). G) CD4^+^ T cells and PD1^+^CTLA4^+^CD4^+^ T cells in malaria patients were analyzed *ex vivo* for the expression of Foxp3 and CD25. Dotplots are shown for one representative patient. The scatter plot shows the frequency of Foxp3^+^ and of Foxp3^+^CD25^+^ cells as percentage of the assessed CD4^+^ and PD1^+^CTLA4^+^ CD4^+^ T cells of all patients included in this analysis (n = 12). H) The mean fluorescence intensity of CTLA4 expression was compared between Foxp3^+^ and Foxp3^-^ CTLA4^+^ CD4^+^ T cells of malaria patients. The dotplot shows the expression of CTLA4 and Foxp3 on CD4^+^ T cells of one representative malaria patient. The scatter plot shows the geometric mean fluorescence intensity of CTLA4 of Foxp3^+^ and of Foxp3^-^CTLA4^+^ CD4^+^ T cells of all patients included in the analysis (n = 12). Horizontal bars represent means. **, P = 0.002 (paired t-test).

We first characterized the expression pattern of coinhibitory receptors, their ligands and regulatory cell markers on T cells during acute *P*. *falciparum* malaria. To this end, we analyzed the blood samples of 31 adult patients with acute *P*. *falciparum* infection in Hamburg, Germany and 19 healthy volunteers via *ex vivo* flow cytometric staining. CD4^+^ T cells of the malaria patients were significantly more likely to express PD1 and CTLA4 than CD4^+^ T cells of the healthy volunteers (P<0.0001; Fig 1A and 1B). The majority of PD1-expressing CD4^+^ T cells also coexpressed CTLA4 (Fig 1A and 1B). Pearson correlation analysis showed that the PD1 expression correlated strongly with CTLA4 expression on CD4^+^ T cells (r = 0.83; Fig 1C) in malaria patients. The expression of CTLA4 and PD1 was transient and dropped after treatment completion and parasite clearance (Fig 1D). There was no correlation between the expression of CTLA4 or PD1 on CD4^+^ T cells and levels of parasitemia of the patient before treatment was initiated (Fig 1E). CTLA4 and PD1 expression on CD4^+^ T cells were also compared between patients with an uncomplicated course of malaria (n = 13) and patients with severe cerebral malaria (n = 3). The percentage of CTLA4^+^CD4^+^ T cell was significantly higher in patients with cerebral malaria than in patients with uncomplicated malaria (Fig 1F). There was a trend towards a more frequent expression of PD1 on CD4^+^ T cells in patients with severe cerebral malaria (Fig 1F).

Flow cytometric analysis showed that the majority of PD1^+^CTLA4^+^CD4^+^ T cells in malaria patients were Foxp3^-^ and CD25^-^, which indicates that they were not Foxp3^+^ natural T_regs_ (nT_regs_) (Fig 1G). Notably, Foxp3^-^CTLA4^+^CD4^+^ T cells expressed significantly higher CTLA4 levels than Foxp3^+^CD4^+^ T cells (3757 versus 2024 geometric MFI, P = 0.002; Fig 1H). Of note, malaria patients also showed a higher percentage of Foxp3^+^CD4^+^ T_regs_ than healthy controls but frequencies of Foxp3^+^ T_regs_ and PD1^+^CTLA4^+^ CD4^+^ T cells did not correlate (S1 Fig).

Not only CD4^+^ T cells, but also more CD8^+^ T cells from malaria patients expressed CTLA4, compared to healthy controls (P<0.001), although the frequencies were much lower than for CD4^+^ T cells (6.5% *versus* 23%, S2 Fig). The malaria and control groups did not differ significantly in terms of the frequency of PD1^+^CD8^+^ T cells although some of the malaria patients showed high PD1 expression on their CD8^+^ T cells (P = 0.06; S2 Fig). Acute malaria not only upregulated CTLA4 and PD1 on the T cells, but also upregulated the ligands for PD1 (PDL1 and PDL2) on CD14^+^ monocytes, B cells and T cells (S4 Fig), relative to the expression patterns in the controls. The CTLA4 ligand CD86 was upregulated on B cells and T cells, but not monocytes (S4 Fig). However, only T cells showed increased expression of CD80, which is an alternative ligand for CTLA4 (S4 Fig).

#### PD1^+^CTLA4^+^CD4^+^ T cells are malaria-specific and have low proliferative potential *in vitro*

**Fig 2:**
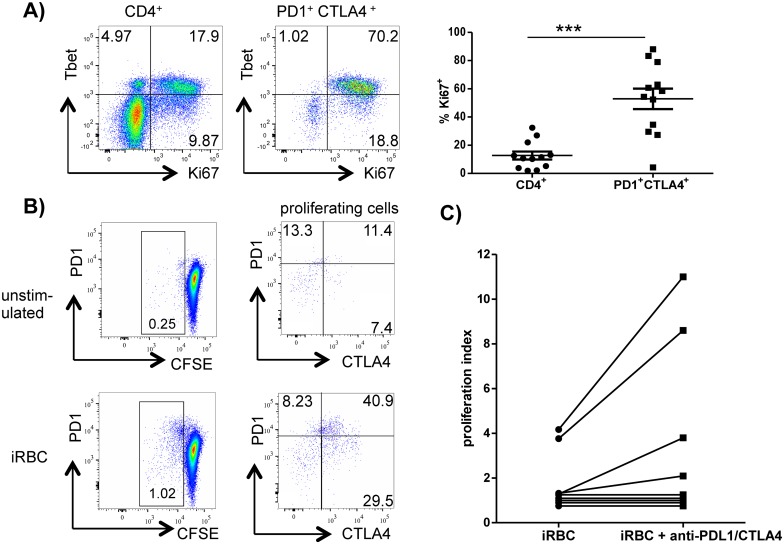
PD1^+^CTLA4^+^ CD4^+^ T cells are malaria-specific. A) Whole blood samples of malaria patients were analyzed for *ex-vivo* expression of intracellular Ki67 and Tbet on CD4^+^ and PD1^+^CTLA4^+^CD4^+^ T cells. Dotplots show one representative donor out of 5. Scatter plots show the frequency of Ki67 expression on the assessed CD4^+^ T cells (left) and PD1^+^CTLA4^+^CD4^+^ T cells (right) for all malaria patients included in the analysis (N = 12). *** P < 0.001. B) PBMC of malaria patients were labeled with CFSE and stimulated with/without iRBC. Cells were first gated for CD4^+^ T cells. Proliferating cells were further analyzed for expression of CTLA4 and PD1. The assay is shown here for one representative malaria patient out of three. The other two patients are presented in S6 Fig. C) PBMC were stimulated with iRBC with/without the addition of 10μg/ml anti-PDL1 and anti-CTLA4 and ^3^H uptakes measured after 120hrs of culture. Proliferation indices were calculated by dividing ^3^H counts of stimulated cells by ^3^H counts of unstimulated cells. A proliferation index > 2 was considered to be a positive response. (n = 11).

The expression of CTLA4 and PD1 on T cells has been associated with an exhausted phenotype in various chronic viral infections, namely, reduced proliferation and cytokine production. This exhausted phenotype recovers when one or both receptors are blocked [13, 29, 30]. We therefore wanted to examine the proliferative potential of PD1^+^CTLA4^+^CD4^+^ T cells in response to plasmodial antigens. To first assess the *in-vivo* activation of PD1^+^CTLA4^+^CD4^+^ T cells in acute malaria, we measured the expression of Ki67, an indirect marker for proliferation. In malaria patients, the percentage of Ki67^+^ cells among the CD4^+^ T cell population was 2–32%, compared to 0.1–4% in the healthy controls (S5 Fig). Closer analysis showed that Ki67 was preferentially expressed by PD1^+^CTLA4^+^CD4^+^ T cells. The frequency of Ki67^+^ cells in the CD4^+^ T cell population was 2–32%, whereas it was 4–88% in the PD1^+^CTLA4^+^CD4^+^ T cell population (Fig 2A). Moreover, five patients were further analyzed for Tbet expression and a large percentage of their Ki67^+^ cells (between 40–89%) coexpressed Tbet, a transcription factor for the Th1 lineage (Fig 2A). This suggests recent activation and proliferation of PD1^+^CTLA4^+^CD4^+^ T cells. However, when peripheral blood mononuclear cells (PBMC) from the malaria patients were stimulated *in vitro* with *P*. *falciparum*—infected red blood cells (iRBC) in CFSE or ^3^H proliferation assays, PBMC of only 40% of the patients exhibited *P*. *falciparum*—specific proliferation (4 of 11 in ^3^H assays and 3 of 6 in CFSE assays). A closer analysis of the CFSE assays revealed that a high frequency (78–90%) of the CD4^+^ T cells that proliferated in response to *P*. *falciparum* antigens *in vitro* expressed CTLA4 or PD1 (Fig 2B and S6 Fig). We therefore asked whether blocking CTLA4 and PD1 would improve these *P*. *falciparum*—specific *in vitro* responses of PBMC. Adding blocking antibodies for CTLA4 and PDL1 enhanced the *in vitro* proliferation of PBMC in 4 of the total 11 patients who were tested (Fig 2C). But overall, the addition of anti-PDL1 and–CTLA4 did not lead to a significant increase of proliferation.

#### PD1^+^CTLA4^+^CD4^+^ T cells coproduce IFNγ and IL10 in response to plasmodial antigens

**Fig 3:**
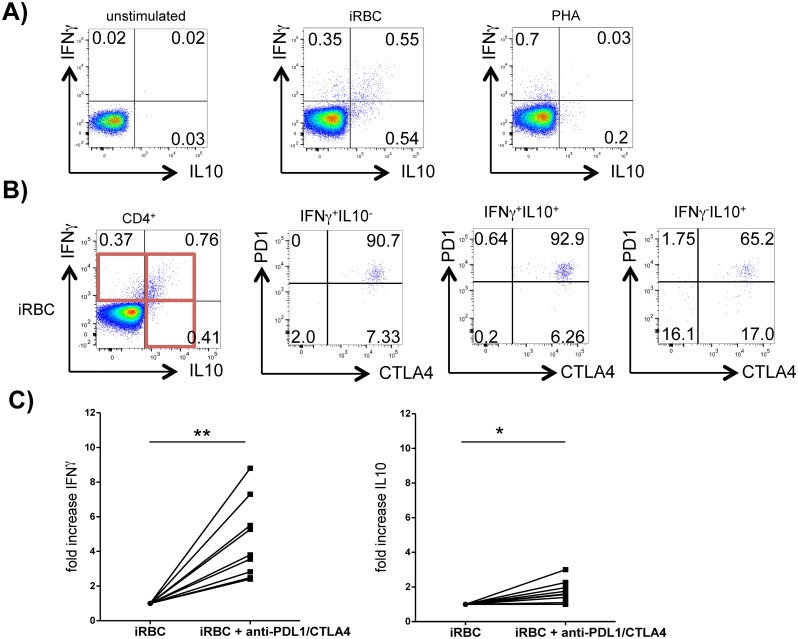
PD1^+^CTLA4^+^CD4^+^ T cells coproduce IFNγ and IL10. PBMC from patients with acute malaria were stimulated with iRBC and addition of Brefeldin A/Monensin for 18h. Stimulation without antigen (medium only) and with uninfected red blood cells (uRBC) served as negative controls. PHA was used as positive control. A) Dotplots show intracellular staining for IFNγ and IL10 in response to medium only (left), iRBC (middle) and PHA (right) after gating for CD4^+^ T cells. One representative donor with acute malaria out of 11 is shown. Gating strategies for cytokine production are shown in S7 Fig. B) IFNγ^+^ and IL10^+^ CD4^+^ T cells were further analyzed for expression of CTLA4 and PD1. One representative patient out of 11 is shown. C) PBMC were stimulated for 120hrs with iRBC and with/without addition of blocking antibodies against CTLA4 and PDL1. IL10 and IFNγ were measured in culture supernatants by ELISA. Fold-increase of cytokine production was calculated by dividing the net iRBC-specific cytokine production with blockade of CTLA4/PDL1 by net-cytokine production without antibody blockade. P = 0.004 (IFNγ) and P = 0.016 (IL10), using the Wilcoxon matched pairs test on net-cytokine concentration results.

We next examined the ability of PD1^+^CTLA4^+^CD4^+^ T cells in malaria patients to produce cytokines in response to plasmodial antigens. Thus, the PBMC of malaria patients were stimulated with iRBC and their production of IFNγ, IL10, TNFα, and IL13 was measured by intracellular staining. *P*. *falciparum*—specific IFNγ and IL10 production was detected in 11 of 17 patients. 0.2% to 2% of the CD4^+^ T cells produced one or both of these cytokines. Interestingly, 40–80% of the IFNγ^+^CD4^+^ T cells coproduced IL10 in response to iRBC stimulation. This coproduction was only observed in response to *P*. *falciparum* but not observed when a polyclonal stimulus (PHA, PMA/Ionomycin, anti-CD3/28) or alternative antigen (CMV) were applied (Fig 3A and S8 Fig). *P*. *falciparum*—specific TNFα and IL13 production was only detected in one of ten patients. Further analysis of the IFNγ^+^ and IL10^+^CD4^+^ T cells showed that the majority of the cells that expressed either or both of these cytokines (40–90%) coexpressed CTLA4 and PD1 (Fig 3B). A high percentage of IFNγ^+^ and/or IL10^+^CD4^+^ T cells (20–60%) also expressed Tbet, indicating Th1 lineage (S8 Fig). Comparing the proportion of IFNγ^+^, IFNγ^+^ IL10^+^ and IL10^+^CD4^+^ T cells in patients with uncomplicated and severe cerebral malaria, we detected a trend towards a decreased proportion of IL10^+^CD4^+^ T cells in patients with severe cerebral malaria (S9 Fig).

Additionally, we analyzed cytokine concentrations in PBMC culture supernatants after 5 days of stimulation with iRBC and assessed how a blockade of CTLA4 and PD1 influenced *P*. *falciparum*-specific cytokine production. Consistent with our flow cytometry results, IFNγ and IL10 were detected in most culture supernatants. Of the 9 patients who were tested, 8 showed *P*. *falciparum*—specific IFNγ production (mean 540 pg/ml, range 30–1477 pg/ml) and 7 showed detectable IL10 production (mean 108 pg/ml, range 30–359 pg/ml). Adding blocking antibodies against CTLA4 and PDL1 strongly enhanced *P*. *falciparum*-specific IFNγ production in all 9 patients who were tested and led to an average 4.5-fold increase of the IFNγ-concentration. The effect on IL10 was less striking with a mean 1.7 fold increase of IL10 concentrations when adding anti-PDL1 and -CTLA4 (Fig 3C).

### PD1^+^CTLA4^+^CD4^+^ T cells regulate other T cells

#### PD1^+^CTLA4^+^CD4^+^ T cells suppress polyclonal and *P*. *falciparum*—specific T cell proliferation

**Fig 4:**
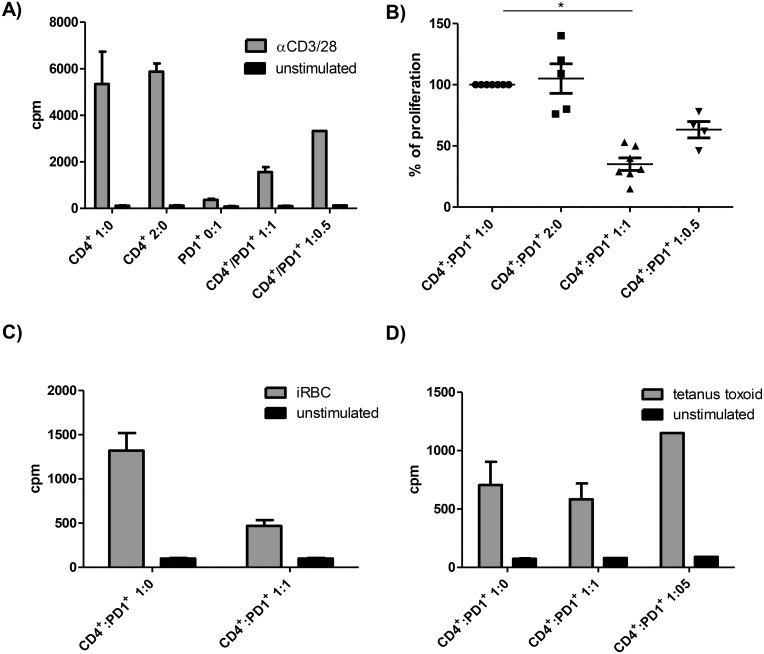
Malaria-induced PD1^+^CTLA4^+^CD4^+^ T cells suppress CD4^+^ T cell proliferation. Cell cultures were set up by stimulating CD4^+^ T cells with anti-CD3/28 (A and B), iRBCs (C) or tetanus toxoid (D) with/without addition of FACS sorted PD1^+^CTLA4^+^CD4^+^ T cells (ratio 1:0, 1:1, 1:0.5). ^3^H-Thymidine uptake was measured after 120 hrs of culture to assess cell proliferation. Cultures were performed in triplicates unless there were insufficient cells, and values represent mean + SE. A) shows the absolute proliferation counts per minute for 2.5 x 10^4^ anti-CD3/28 stimulated CD4^+^ T cells (ratio 1:0), 5x 10^4^ CD4^+^ T cells (ratio 2:0), 2.5 x 10^4^ PD1^+^CTLA4^+^CD4^+^ T cells (0:1) and 2.5 x 10^4^ CD4^+^ T cells with equal (ratio 1:1) or half the numbers (ratio 1:0.5) of PD1^+^CTLA4^+^CD4^+^ T cells for one representative malaria patient out of 7. Adding twice the number of CD4^+^ T cells, 5 x 10^4^ cells, (2:0), was included as a control condition to exclude higher cell numbers as a cause for suppressed cell proliferation. B) summarizes the results for all malaria patients included in this experiment (n = 7 for condition 1:0 and 1:1). The net (stimulated minus spontaneous) proliferation counts for 2.5 x 10^4^ CD4^+^ T cells (1:0), stimulated with anti-CD3/28, were set as 100%. Proliferation results for other cell culture conditions are expressed as percentage of net proliferation counts of CD4^+^ T cells (ratio 1:0). Statistical analysis was conducted using the Wilcoxon matched pairs test on net counts per minutes (*, P = 0.016). C and D) For antigen-specific stimulation, 10^5^ CD4^+^ T cells were stimulated with equal numbers of irradiated feeder cells and with/without equal numbers of PD1^+^CTLA4^+^CD4^+^ T cells. The diagrams show one representative suppression assay of 2, using iRBC (C) or tetanus toxoid as specific antigen (D).

PD1 and CTLA4 both negatively regulate T cell activation, by competition with the co-stimulatory CD28 for shared ligands [31] and downregulation of cell effector functions upon ligation [32]. However, both receptors, CTLA4 and PD1, have also been associated with regulatory T cell populations [33]. We therefore asked whether the malaria-induced PD1^+^CTLA4^+^CD4^+^ T cells could suppress other CD4^+^ T cells. To this end, we isolated PD1^+^CTLA4^+^CD4^+^ T cells based on surface expression of PD1. To obtain this population, CD4^+^ T cells were negatively selected with immunomagnetic beads and natural T_regs (_nT_regs)_ were removed by FACS sorting on the basis of their CD25^+^CD127^dim^ expression profile. We then selected CD4^+^ T cells with high surface levels of PD1 (the gating strategy of selected cells are shown in S10 Fig). Post-selection analyses of purity revealed that 50–95% of the selected cells were PD1^+^CTLA4^+^CD4^+^ T cells. This PD1^+^CTLA4^+^CD4^+^ T cell population proliferated poorly when subjected to polyclonal stimulation with anti-CD3/28 (Fig 4A). Interestingly, when the CD4^+^ T cell population was co-cultured with the isolated PD1^+^CTLA4^+^CD4^+^ T cell population at a 1:1 ratio, the anti-CD3/28 induced proliferation of the CD4^+^ T cells dropped on average by 65% (50–85%) (Fig 4A and 4B). This suppressive effect was dose-dependent when lower numbers of PD1^+^CTLA4^+^CD4^+^ T cells were added (i.e. ratio 1:0.5) (Fig 4A). Of note, due to the low cell recovery of sorted PD1^+^ T cells (between 50 000–600 000 cells per patient), a detailed cell-based analysis such as CFSE assays could not be conducted. We included control conditions with twice the number of CD4^+^ T cells (2:0) or add back of sorted PD1^-^CD4^+^ T cells to exclude that higher number of cells per well or non-specific effects of the cell sorting process led to the observed suppression of proliferation by PD1^+^CTLA4^+^CD4^+^ T cells. No suppression was observed in these control culture conditions (Fig 4A and S11 Fig). We also compared the suppressive effect of the isolated PD1^+^CTLA4^+^CD4^+^ T cells with CD25^+^CD127^dim^ nT_regs_. The observed suppression of CD4^+^ T cells proliferation by nT_regs_ was stronger than by PD1^+^CTLA4^+^CD4^+^ T cells (77–89% compared to 50–85%, S11 Fig).

We next examined the effect of PD1^+^CTLA4^+^CD4^+^ T cells on *P*. *falciparum*—specific CD4^+^ T cell responses. Adding PD1^+^CTLA4^+^CD4^+^ T cells to complete CD4^+^ T cells cultures stimulated with iRBC in the presence of irradiated feeder cells suppressed *P*. *falciparum—*specific T cell proliferation (Fig 4C). However, this suppressive effect was not observed with the alternative antigens tetanus toxoid or CMV (Fig 4D and S11 Fig). In summary, these data show that malaria-induced PD1^+^CTLA4^+^CD4^+^ T cells inhibit the proliferation of other T cells in a cell extrinsic manner. However, that suppressive effect is limited to malaria-specific and polyclonal stimulation of PD1^+^CTLA4^+^CD4^+^ T cells.

#### Suppression by PD1^+^CTLA4^+^CD4^+^ T cells requires contact with the target T cell and does not depend on CTLA4, PD1, TGFβ or IL10

**Fig 5:**
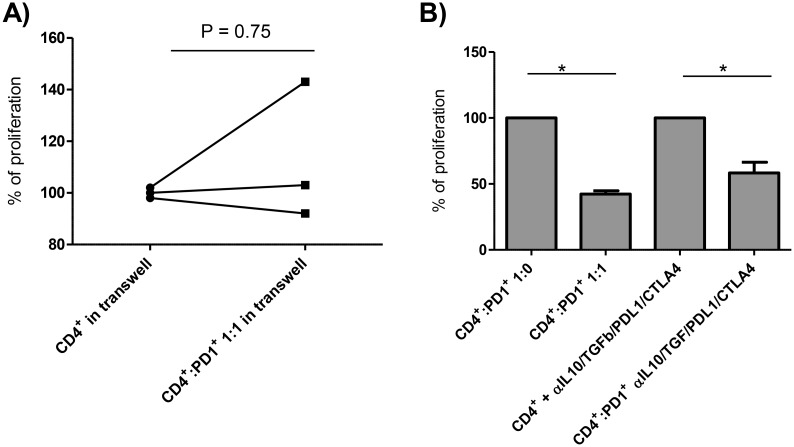
PD1^+^CTLA4^+^CD4^+^ T cell-mediated suppression requires cell contact. To determine mechanisms involved in the suppression mediated by PD1^+^CTLA4^+^CD4^+^ T cells, we conducted suppression assays in transwell systems and in the presence of blocking antibodies for IL10R, TGFβ, CTLA4 and PDL1. The net (stimulated minus spontaneous) proliferation counts for CD4^+^ T cells (ratio 1:0), stimulated with anti-CD3/28, were set as 100%. Proliferation results for other cell culture conditions are expressed as percentage of net proliferation counts of CD4^+^ T cells (ratio 1:0). A) 5 x10^4^ CD4^+^ T cells were stimulated with anti-CD3/28 and cultured with/without an equal number of PD1^+^CTLA4^+^CD4^+^ T cells, separated by a 0.2μm transwell membrane. P = 0.75, using a Wilcoxon matched pairs test (n = 3). B) 2.5 x10^4^ CD4^+^ T cells were stimulated with anti-CD3/28 and cultured with/without an equal number of PD1^+^CTLA4^+^CD4^+^ T cells (abbreviated with PD1^+^) and with/without addition of 10μg/ml blocking antibodies for IL10R, TGFβ, CTLA4 and PDL1 (n = 4). *, overall P = 0.01. Statistical significance was determined using a Friedman test with Dunn´s multiple comparisons test.

A transwell system was used to elucidate the inhibitory mechanisms of the PD1^+^CTLA4^+^CD4^+^ T cells. All patients included in the transwell experiment (n = 3) showed strong suppression when CD4^+^ T cells and sorted PD1^+^CTLA4^+^CD4^+^ T cells were co-cultured (Fig 4B). But the suppressive effect of the PD1^+^CTLA4^+^CD4^+^ T cells on anti-CD3/28-induced proliferation of the purified CD4^+^ T cells was completely abrogated when the two cell populations were separated by transwell inserts (Fig 5A). Since the PD1^+^CTLA4^+^ T cells suppressed the proliferation of purified CD4^+^ T cells to anti-CD3/28 beads in the absence of antigen-presenting cells (Fig 4A and 4B), we can conclude that the inhibitory function of the PD1^+^CTLA4^+^CD4^+^ T cells requires direct T-T cell interactions. As the blockade of CTLA4 and PD1/PDL1 enhanced the proliferative response of PBMC to *P*. *falciparum* in some malaria patients (Fig 2C), we used these blocking antibodies to assess whether these coinhibitory receptors participate in the suppressive role of PD1^+^CTLA4^+^CD4^+^ T cells. We also added blocking antibodies to the anti-inflammatory cytokines IL10 and TGFβ. However, these blocking antibodies in combination did not reverse the ability of the PD1^+^CTLA4^+^CD4^+^ T cells to suppress the proliferation of the CD4^+^ T cells (Fig 5B). Thus, other mechanisms, including cell-cell contact, are required for the *in vitro* suppressive effect of PD1^+^CTLA4^+^CD4^+^ T cells.

## Discussion

The present study explored the role of coinhibitory receptors as an immune regulatory pathway in acute *P*. *falciparum* malaria. We initially hypothesized that the induction of coinhibitory receptors, primarily CTLA4 and PD1, is an important and adaptable mechanism by which CD4^+^ T cell responses in malaria are downregulated. Indeed, we found that the majority of *P*. *falciparum*-specific CD4^+^ T cells expressed both CTLA4 and PD1 and that blocking both receptors enhanced their *P*. *falciparum*-specific effector responses. Surprisingly, subsequent experiments showed that the PD1^+^CTLA4^+^CD4^+^ T_eff_ cells in malaria patients had an acquired extrinsic regulatory function by which they suppressed the *P*. *falciparum*-specific and polyclonal proliferation of other T cells. These PD1^+^CTLA4^+^CD4^+^ T_eff_ cells therefore constitute a distinct regulatory T cell population which is induced during acute malaria.

Our collection of blood from adult patients with imported *P*. *falciparum* malaria in Hamburg, Germany, enabled us to conduct complex functional experiments in the setting of acute infection and disease that would not be possible in holoendemic areas, where adults rarely develop acute febrile malaria and only small amounts of blood can be collected from sick children. Our patient cohort consisted largely of two groups, namely, Caucasian patients who had malaria for the first time, and patients of West African descent who reside in Germany and whose last treatment for malaria was at least 5 years before the index infection (S1 Table). Notably, we did not observe a difference between the two main groups of our patient cohort in terms of their T cell phenotypes and regulatory function (S12 Fig).

Recently, several studies have observed that patients with acute malaria, and children in endemic regions, who are exposed to *P*. *falciparum*, express higher levels of CTLA4 and PD1 on their T cells than uninfected or unexposed control subjects [24, 26–28]. This increased expression has been proposed to be a sign of T cell exhaustion. Indeed, when coinhibitory receptors are blocked, T cells from patients with *Plasmodium vivax* malaria produce higher cytokine levels in response to parasite antigens [28]. However, whether these receptors induce a similar downregulation of the T cell response to *P*. *falciparum* malaria had not been addressed so far.

Our study showed that during acute *P*. *falciparum* malaria, high numbers of CD4^+^ T cells expressed CTLA4 and PD1 and mostly coexpressed both receptors. Although both, CTLA4 and PD1, are rapidly upregulated after T cell receptor-mediated activation, their expression can also be upregulated by alternative cytokine-based mechanisms [34, 35]. While clearly the vast majority of *P*. *falciparum*-specific T cells in our study were CTLA4^+^ and PD1^+^, it was not possible to determine if all PD1^+^CTLA4^+^CD4^+^ T cells were specific for *P*. *falciparum*-antigens or whether bystander activation contributed to the high numbers of CTLA4^+^ and PD1^+^ T cells. Importantly, patients with severe cerebral malaria showed a higher frequency of CTLA4^+^CD4^+^ T cells than patients with uncomplicated malaria. This analysis was restricted by the low number of patients with cerebral malaria (n = 3) included in our study and needs to be further validated with higher numbers of patients with severe malaria. But our data support results from an earlier study with children in Ghana, in which a higher percentage of CTLA4^+^CD4^+^ T cells was observed in children with severe malaria, predominantly severe anemia, than in children with uncomplicated malaria [36]. These observations suggest that a dysregulation of the CD4^+^ T cell function is associated with an increased risk for severe complications of malaria.

By contrast, we found that the degree of parasitemia at time of diagnosis did not correlate with the frequency with which CD4^+^ T cells expressed CTLA4 or PD1. This is different from observations in chronic and acute viral diseases where it has been shown consistently that viral load correlates with PD1 and CTLA4 expression on T cells [14, 37–39]. It should be noted that peripheral parasitemia does not reflect the numbers of sequestered parasites in the microcirculation and is therefore a poor predictor of complete parasite biomass [40]. Besides, peak parasitemia and highest frequencies of CTLA4^+^ and PD1^+^CD4^+^ T cells might occur at later time points than time of diagnosis (Fig 1D). Thus, a correlation between CTLA4 and PD1 expression and complete parasite biomass or peak parasitemia cannot fully be excluded. Interestingly, a study by Schlotmann *et al*. detected a correlation between CTLA4 expression and peak parasitemia (but not parasitemia at time of diagnosis) in adult patients with acute malaria [26]. However, it is also conceivable that the lack of correlation between parasitemia and CTLA4 and PD1 expression in our study reflects the fact that the parasite-induced upregulation of coinhibitor expression is readily saturated by antigen-load and cannot be further increased by higher parasitemia. Other factors such as the duration of antigen exposure or cytokine levels might additionally influence the CTLA4 and PD1 expression in malaria.

To date, several studies have reported that T cell responses to plasmodial and other antigens are reduced during acute malaria and recover after treatment and convalescence [41–45]. Several mechanisms underlying this phenomenon have been proposed, including impairment of antigen-presenting cell function, the production of anti-inflammatory cytokines, and the induction of apoptosis [45, 46]. Similarly, we found in our study that only 40% of our patients showed measurable T cell proliferation and 65% produced cytokines (predominantly IFNγ and IL10) by intracellular staining in response to *P*. *falciparum*, respectively. We propose that the strong induction of CTLA4 and PD1 contributes to the reduced effector function of *P*. *falciparum*-specific T cells during acute malaria. Blocking both, CTLA4 and PDL1, enhanced the T cell proliferation in 4 of 11 malaria patients. The blockade showed a stronger effect on cytokine production and increased the malaria-specific IFNγ and IL10 production of all patients who exhibited a cytokine response to *P*. *falciparum* and demarcated a positive IFNγ response in one patient who was priorly unresponsive. Our results corroborate observations by us and other groups in rodent malaria models that administration of antibodies against CTLA4 and/or PDL1 *in vivo* increases the proinflammatory T cell responses in murine malaria [18, 23, 24, 47]. Moreover, a recent study showed that blocking coinhibitory receptors, including PDL1 and CTLA4, enhanced cytokine responses to *P*. *vivax* antigens *in vitro* [28]. Of note, we observed a small but significant increase of the frequencies of PDL2^+^ macrophages, T cells and B cells. Further enhancement of the T cell effector function might be achieved with additional blockade of the PD1/PDL2 axis [48], which has not been examined in malaria so far.

Taken together, our data support the notion that the observed dysfunction of *P*. *falciparum*-specific T cells during acute malaria can be attributed, at least in part, to the upregulation of CTLA4 and PD1.

PD1^+^CTLA4^+^CD4^+^ T cells were the main source of *P*. *falciparum*-specific cytokines and predominantly produced IFNγ and IL10. Most coproduced both of these cytokines. Both, IFNγ and IL10, play critical roles in the immune homeostasis in acute malaria. IFNγ is needed to activate phagocytosis and control parasitemia, while IL10 is crucial for counterbalancing the inflammatory response [49–52]. While IL10 can be produced by various cell populations, including B cells, monocytes, NK cells, Th2-type T cells, and Type 1 T_regs_ (Tr1 cells), several studies suggest that IFNγ^+^IL10^+^ Th1 cells are a particularly important source of IL10 in human and murine parasitic infections [9, 50, 53]. Importantly, IFNγ^+^IL10^+^ Th1 cells have been shown recently to prevent immunopathology in rodent models of toxoplasmosis and malaria [10, 49]. Similarly, studies in endemic areas identified IFNγ^+^IL10^+^ Th1 cells in children regularly exposed to or infected with *P*. *falciparum* [51, 53, 54]. Moreover, low frequencies of these cells were associated with the development of severe malaria in children in the Gambia [11]. Importantly, we also noticed a trend towards a decreased IL10^+^ to IFNγ^+^ T cell ratio in our patients with severe cerebral malaria compared to patients with uncomplicated malaria but our sample size of patients with severe cerebral malaria was low (n = 3). Specifically, there was a lower proportion of IL10 single-positive Th1 CD4^+^ T cells. This is different to the prior study in Gambia, where a polycloncal stimulus with PMA/Ionomycin was used. High ratios of TNFα and INFγ levels to IL10 or TGFβ levels have also been observed in other studies in patients with severe malaria [55, 56].

Taken together, our data showed that IFNγ^+^IL10^+^ CD4^+^ T cells are present during the first-ever symptomatic infection with *P*. *falciparum* in naïve individuals as well as in individuals who were last exposed > 5 years ago. Repeated exposure is therefore not required for the induction of IFNγ^+^IL10^+^ CD4^+^ T cells. Notably, we also observed that both the IFNγ^+^IL10^+^ cells and even a substantial percentage of the IL10 single-positive cells expressed Tbet, suggesting that they derive from Th1 lineage. This is consistent with *in vitro* studies employing the complement factor CD46 that showed Th1 effector cells acquire the ability to produce IL10 and convert from IFNγ^+^ into IFNγ^+^IL10^+^ cells during ongoing stimulation before terminally transitioning to IL10 single-positive Th1 CD4^+^ T cells. The latter cells exert a suppressive function and have a Tr1-like phenotype [57]. It is highly likely that the strong antigen exposure and the cytokine milieu during acute malaria activates the IFNγ to IL10 switch, which serves as a negative feedback loop to prevent overwhelming inflammation and tissue damage. This concept is further supported by the observation by us and others that IFNγ to IL10 ratios might be altered in patients with severe malaria.

Interestingly, isolated PD1^+^CTLA4^+^CD4^+^ T cells acted like antigen-specific Tr1-like cells: they suppressed the *P*. *falciparum*-specific and anti-CD3/28-induced proliferation of the CD4^+^ T cell population from malaria patients. However, they did not suppress the responses to other antigens such as tetanus toxoid. This suggests strongly that PD1^+^CTLA4^+^ T cells are *P*. *falciparum*-specific and require prior activation to exert their cell-extrinsic suppressive function. This activation can be mediated by a polyclonal stimulans such as anti-CD3/28 or by their specific *P*. *falciparum* antigen. The PD1^+^CTLA4^+^ T cells in our malaria patients were clearly distinct from natural T_regs_ as they did not express Foxp3 or CD25, nor were they CD49b^+^LAG3^+^ (S13 Fig), which are recently identified markers for Tr1 cells [58]. However, the expression of both receptors, CTLA4 and PD1, has repeatedly been associated with a regulatory phenotype. In particular, the CTLA4 expression of our PD1^+^CTLA4^+^CD4^+^ T cells was significantly higher than in Foxp3^+^ natural T_regs_. This is interesting because although CTLA4 was originally discovered as a cell-intrinsic inhibitor, there is some evidence that conventional CTLA^+^ T cells can play a cell-extrinsic regulatory function but current observations are restricted to murine experiments [59–61]. PD1 expression on the other hand associates with IL10-producing Tr1 or Tr1-like T_regs_ [62, 63]. Indeed, we found that the PD1^+^CTLA4^+^CD4^+^ T_eff_ cells in our malaria patients produced the regulatory cytokine IL10 in response to *P*. *falciparum*-antigens. But unlike the inhibitory function of classical Tr1 cells, the suppressive effect of the PD1^+^CTLA4^+^CD4^+^ T_eff_ cells was not abrogated by blocking IL10. In fact, even the combined blockade of CTLA4, PDL1, TGFβ, and IL10 did not alter the suppressive function of the PD1^+^CTLA4^+^CD4^+^ T_eff_ cells. Expression of coinhibitory receptors and the cytokines TGFβ and IL10 are important tools to maintain immune homeostasis [18, 23, 24, 47, 49, 64]. But the observed suppressive effect of PD1^+^CTLA4^+^CD4^+^ T_eff_ cells is independent of these regulatory mechanisms. Transwell experiments revealed that the suppression was dependent on T-T cell contact, similar to what is seen in nT_regs_. It is highly possible that the suppressive effect of the PD1^+^CTLA4^+^CD4^+^ T_eff_ cells is mediated by several mechanisms and not one single mechanism can be identified, as has been shown in nT_regs_. Possible mechanisms include release of granzyme B and perforin, disrupting the metabolic state of T cells via the production of the ectoenzymes CD39 and CD73 or consumption or downregulation of IL2 [65]. The fact that the PD1^+^ CTLA4^+^ CD4^+^ T_eff_ cells expressed only low levels of CD25 argues against consumption of IL2 as a possible mechanism underlying their suppressive activity. But further studies that determine how PD1^+^ CTLA4^+^CD4^+^ T cells suppress other T cells are clearly warranted. Mouse models of malaria are probably a powerful tool to further investigate the suppressive mechanisms.

To our knowledge, this is the first time a population of antigen-specific T_eff_ cells with a cell-extrinsic suppressor function has been reported in an acute infection in humans. Our observations partially support previous findings by Häringer *et al*., who identified a population of IFNγ^+^IL10^+^ effector-like T cells with regulatory function in the blood of healthy volunteers [62]. Similar to our study, these cells express high levels of PD1 and CTLA4 and low levels of CD127 and respond to persisting antigens such as *Candida* and CMV but not to vaccine antigens. However, the suppressive activity of these IFNγ^+^IL10^+^ T cells mainly depends on IL10. Several other studies have described populations of allergen-specific PD1^+^CTLA4^+^IL10^+^ T cells in the peripheral blood but their suppressive function *in vitro* depends on multiple factors, including CTLA4, PD1, and IL10 [33, 63]. Interestingly, a study by Che *et al*. showed that naïve T cells failed to expand *in vitro* in the presence of HIV-primed T cells, and that this suppressive effect was also dependent on T-T cell contact [66].

While peripheral control of inflammation has generally been attributed to adaptive Tr1 cells, which are thought to be a distinct lineage, our observations raise the intriguing hypothesis that during an acute infection, regulatory function is transiently acquired by T_eff_ cells to control the T cell response by three mechanisms: a) autoregulation of the T_eff_ response through upregulation of CTLA4 and PD1 which inhibit cytokine production b) cytokine switch from IFNγ to IL10 production and c) cell-extrinsic inhibition of the T cell proliferation.

This possibility is potentially exciting given the increasing focus on immune-modulating therapies in the last few years that has led to the licensing of antibodies against coinhibitory receptors and the first trials on the transfer of T cell populations [67, 68]. Modulating T_eff_ cells and their regulatory pathways could offer new and exciting approaches for preventing immune-mediated pathologies in severe infections or for modulating vaccine responses in the future. Further studies that elucidate the factors involved in the induction of PD1^+^ CTLA4^+^ T cells, their effector and regulatory functions, and their immunological memory are strongly warranted. Studies that explore the relationship between the regulatory pathways and the outcome of *P*. *falciparum* infection in humans in endemic areas would also be very valuable.

In conclusion, our results shed further light on the complex immune regulation in acute malaria. We conclude that PD1^+^CTLA4^+^ T_eff_ cells play a crucial role in the immune regulation: they arise from Th1 effector cells and acquire a suppressive phenotype, by expressing coinhibitory receptors, switching to IL10 production and by cell-extrinsic suppression of other T cells. Such a negative feedback loop is probably not unique for *P*. *falciparum* malaria and could be a common autoregulatory mechanism that serves to control overwhelming Th1 responses and prevent immunopathology in acute infections.

## Materials and Methods

### Study population

In total, 25 patients with acute malaria were enrolled during their inpatient treatment at the University Hospital Eppendorf in Hamburg between the first and sixth day of the treatment. Patients were enrolled consecutively between March 2013 and September 2014. The clinical characteristics of the enrolled patients are summarized in S1 Table. The majority of these patients (14 of 25) were of West African origin and had been living in Germany for ≥5 years (5–20 years). 8 of the remaining patients were Germans who were infected during vacation or short-term work assignments in a malaria endemic country. Two were German expatriates, living in Western Africa and one patient from Gambia was visiting Germany on vacation. Patients of West African origin had suffered from repeated malaria episodes as children but had not been diagnosed with malaria > 5 years. The 25 enrolled patients were on average 42 (range 18–70) years old. All had microscopically detectable parasitemia and five had severe malaria, according to the definitions of the WHO [69]. Of the five patients with severe malaria, three suffered from cerebral malaria and two were diagnosed with hyperparasit emia. Patients with cerebral malaria were treated with Artesunate iv, followed by atovaquone-proguanil. All other patients were treated with artesiminin-combination therapy (dihydroartemisin-piperaquin) or atovaquone-proguanil p.o.. Between 5 and 50 ml of heparinized blood was collected from each malaria patient and used for flow cytometric analysis and functional assays. Due to limitations on number of cells, only a subset of functional assays and/or flow cytometric staining could be carried out for each blood sample. In addition to the enrolled patients, anonymous blood samples (1 ml each) from 15 adult patients with an active *P*. *falciparum* infection were obtained from the clinical diagnostic lab of the Bernhard Nocht Institute of Tropical Medicine. Baseline information such as age, parasitemia and presentation of symptoms were available for these patients. The latter samples were only subjected to *ex vivo* flow cytometric T cell analysis (Fig 1B, 1C and 1E). For a small number of patients, additional follow-up samples were received during treatment via the diagnostic lab and used for *ex vivo* flow cytometric T cell phenotyping. The 19 control subjects were healthy adult staff members of the Bernhard Nocht Institute of Tropical Medicine and the University Medical Centre Hamburg Eppendorf.

### Determination of *P*. *falciparum* infection

*P*. *falciparum* infection was determined microscopically by experienced lab technicians at the Bernhard Nocht Institute of Tropical Medicine. Thick and thin blood smears were stained with 4% Giemsa and examined under oil immersion (original magnification × 100).

### Cell phenotyping by flow cytometric analysis

*Ex vivo* staining for immunophenotypic analysis was conducted on fresh whole blood samples from malaria patients and healthy volunteers. For malaria patients, samples were included on the day of diagnosis (day 1) prior to treatment whenever available. Alternatively, the first blood sample obtained from that specific patient was included for flow cytometric analysis. Thus, 100 μl of fresh whole blood was incubated for 30 min at 4°C with the following antibodies: CD4 BV510 (clone OKT4), CD8 AF700 (RPA-T8), CD14 AF 700 (HCD14), CD19 BV510 (HIB19), CD11c PECy7 (Bu15), CD45RA FITC (HI100), CCR7 PECy7 (G043H7), PD1 PerCP Cy5.5 (EH12.2H7), BTLA Biotin (MIH26) with Streptavidin-BV 421, CD25 PE Cy7 (BC96), CD127 BV 421 (A019D5), PD-L1 APC (29E.2A3), PD-L2PE (24F.10C12), CD80 BV 421 (2D10), and CD86 AF488 (IT2.2) (all from BioLegend). Thereafter, the blood samples were lysed and fixed using a Lysis/Fixation buffer (BioLegend), and washed. Subsequent intracellular staining for CTLA4 PE (L3D10), Foxp3 AF647 (259D), Tbet APC (4B19), Granzyme B AF647 (GB11), CD3 APC Cy7 (SK7) (all from BioLegend), and Ki67 AF 488 (MK167) (eBioscience) was conducted using the eBioscience Foxp3/Transcription factor staining buffer set according to the manufacturer’s protocol. The flow cytometric data were collected on a LSR II Cytometer (4 laser, Becton Dickinson) and analyzed using FlowJo software (Treestar). Compensations were set using single stained/unstained beads. At least 100 000 cells were collected in the lymphocyte gate. Cells were first gated on forward/sideward scatter to exclude cell debris and gates were set for lymphocytes and monocytes. T cells were then gated on the basis of the lineage markers CD3, CD4 and CD8. Monocytes/macrophages were defined as CD14^+^CD3^-^ cells. B cells were defined as CD19^+^CD3^-^ cells. Fluorescence minus one (FMO) controls were used to define subsequent gates for coinhibitory receptors and ligands and regulatory, memory, and activation markers. FMO is a staining control that includes all antibodies used in a flow cytometric assay except for one fluorochrome of interest (termed FMO) to control for the contribution of spectral overlap to the background when using multiple fluorochromes [70, 71].

### Antigens and mitogens

*In vitro P*. *falciparum* antigen-specific CD4^+^ T cell proliferation and cytokine expression were measured using *P*. *falciparum*-infected red blood cells (iRBC) as the antigen. For this, *P*. *falciparum* cultures (strain 3D7) were maintained in 0^+^ erythrocytes in RPMI-1640 medium supplemented with 0.5% AlbuMAX II (Invitrogen) at 37°C according to standard methods [72]. Schizont and late trophozoite stage parasites were isolated magnetically using the Miltenyi LD column. The percentage of infected erythrocytes after magnetic isolation was typically 80–90%. The iRBC were stored in PBS at -70°C until use in cell culture at two iRBC per T cell/PBMC. Uninfected red blood cells (uRBC), namely, the 0^+^ erythrocytes from the same donors that were used for the parasite cultures, served as negative controls at two uRBC per T cell/PBMC. CMV pp65 protein (Miltenyi, 1 μl/ml) and tetanus toxoid (10 μg/ml) served as alternative antigens. For polyclonal stimulation in proliferation assays, 1 μg/ml soluble anti-CD3 (clone UCHT1, eBioscience) and 1 μg/ml anti-CD28 (clone CD28.2., eBioscience) were used. Alternatively, anti-CD3/28-coated beads (Dynal T cell expander) were used at a ratio of five cells/bead. PHA (Sigma-Aldrich at 5 μg/ml) or PMA/Ionomycin (50ng/500ng) served as the positive controls for intracellular cytokine staining.

### Cell isolation and sorting

PBMC were isolated from heparinized whole blood within 2 hours of collection by standard density gradient centrifugation with Ficoll Paque (GE Healthcare) using the SepMate System (Stemcell technologies) according to the manufacturer’s instructions. The isolated PBMC were then directly subjected to antigen/mitogen stimulation assays that measured cell proliferation and cytokine production by intracellular staining or underwent further T cell isolation steps. For cell cultures, fresh PBMC were resuspended in complete RPMI (cRPMI), consisting of RPMI 1640 (PAA laboratories) supplemented with 10% pooled human AB serum (Sigma-Aldrich), 4 mM L-glutamine, 25 mM HEPES, and 80 mg/ml gentamicin (both PAA laboratories). For isolation of T cell subsets, freshly isolated PBMC were washed and resuspended in MACS buffer (PBS with 0.5% BSA and 2 mmol/l EDTA). The PBMC were then negatively selected for CD4^+^ T cells using the CD4^+^ T cell isolation kit from Miltenyi according to the manufacturer’s protocol. The isolated CD4^+^ T cell preparations were routinely >90% pure. An aliquot of each CD4^+^ T cell preparation was set aside for cell culture. The remaining CD4^+^ T cells were resuspended in PBS supplemented with 1% human AB serum and surface-stained with anti-CD4 APC (RPA-T4), anti-CD25 PECy7 (BC96), anti-CD127 AF 488 (A019D5), anti-PD1 PerCP Cy5.5 (EH12.2H7), and anti-CTLA4 PE (L3D10). Gates were set based on FMO controls. Natural T_regs_ were first excluded on the basis of high expression of CD25 and low expression of CD127. The remaining CD4^+^ T cells were then sorted into PD1^+^ CD4^+^ T cells and PD1^neg^ CD4^+^ T cells. The PD1^+^ T cell fractions were 50–95% pure for PD1 and CTLA4 after conducting intracellular staining for CTLA4 (S10 Fig). The CD4^-^ cells that were acquired in the magnetic-bead sorting step were irradiated with 3000 rad and served as antigen-presenting cells. After isolation, the cells were resuspended in cRPMI and used for cell culture.

### Cell culture and suppression assays

Freshly isolated PBMC or T cells were cultured in round-bottom 96-well plates directly after isolation. To assess antigen-specific responsiveness of PBMC, the cells were cultured at 1×10^6^/ml for 120 hours with 2 iRBC or uRBC/cell, after which 80 μl of supernatant was removed for cytokine detection assays. To assess whether blocking the ligation of PDL1 and CTLA4 would enhance the proliferation and cytokine production of these PBMC, the cells were coincubated with 10 μg/ml anti-PDL1 (MIH1, eBioscience) and 10 μg/ml anti-CTLA4 (Ipilimumab). Isotype controls for human and mouse IgG1 (BioLegend ET901 and eBioscience P3.6.2.8.1) were used at 10μg/ml. To assess the ability of PD1^+^CTLA4^+^CD4^+^ T cells to suppress other CD4^+^ T cells, 1.25×10^5^ CD4^+^ T cells/ml were stimulated with anti-CD3/28 beads (5 cells/bead ratio). PD1^+^CTLA4^+^CD4^+^ T cells were added to the cultures at different CD4^+^/ PD1^+^ ratios, namely, 1:0; 1:1; and 1:0.5. For antigen-specific suppression assays, CD4^+^ T cells were cultured at 5×10^5^ T cells/ml with iRBC, uRBC, CMV, or tetanus toxoid with or without PD1^+^CTLA4^+^CD4^+^ T cells in the presence of a final concentration of 5×10^5^/ml irradiated CD4^-^ cells, which served as antigen-presenting cells. After 120 hours of culture, 80 μl of supernatant was removed for cytokine detection assays. Lymphocyte proliferation in the PBMC and suppression assays was determined by pulsing the cultures after 120 hours with 1 μCi/ml ^3^H-Thymidine for 18 hours, harvesting the cells onto cellulose filters, and measuring radiolabeled thymidine incorporation by scintillation counting. The results were expressed as counts per minute (cpm). A stimulation index >2 (mean cpm of stimulated cells/mean cpm of unstimulated cells) was considered to be a positive proliferative response. To determine the suppressive mechanism, the suppression cultures were co-cultured with anti-PDL1 (MIH1, eBioscience), anti-CTLA4 (Ipilimumab), anti-TGFβ (1D11.16.8, eBioscience), and anti-IL10R (3F9, BioLegend) (all at 10 μg/ml). Transwell experiments were conducted using Nunc transwell inserts. Cell culture conditions were set up in triplicates when sufficient cell numbers were available. However, limitations in cell numbers meant that not all samples could be subjected to all experimental conditions.

### CFSE proliferation assay

Freshly isolated PBMC were labeled with 15 mM CFSE (Molecular Probes) and cultured with iRBC, uRBC or medium (negative controls), or anti-CD3/28 (positive control), harvested after 72 hours, surface-stained for CD4, CD8, CD3 and PD1, and intracellularly stained for CTLA4 using the eBioscience Foxp3/Transcription factor staining buffer. Dead cells were excluded using the UV live/dead stain (Life Technologies).

### Intracellular staining for cytokines

To detect intracellular cytokines, PBMC were cultured at 10^6^ cells/ml and stimulated with iRBC, uRBC, PHA, anti-CD3/28, CMV or without antigen for 18 hours. Brefeldin A and Monensin (1 μl/ml each, both from BioLegend) were added after 6 hours of culture. For control cultures with PMA/Ionomycin, PBMC were stimulated with 50ng PMA and 500ng Ionomycin for a total duration of 12h. The cells were then washed and surface-stained with the following antibodies: CD4 BV 510, CD8 AF 700, PD1 PerCP Cy5.5, and CD127 BV 421. To detect dead cells, the cells were also stained with UV live/dead (Life Technologies). The cells were then fixed and permeabilized using the eBioscience Foxp3 /Transcription factor staining buffer set and intracellularly stained using the following antibodies: IFNγ FITC (4S.B3), IL10PE (JES3-9D7), IL4BV 421 (MP4-25D2), TNFα PECy7 (MAB11), IL13PE (JES10-5A2), CTLA4 PE (L3D10), Foxp3 AF 647 (259D), CD3 APC Cy7 (SK7) and Tbet APC (4B19). Samples were acquired on a LSR II and analyzed using FlowJo software. Gates were set on the basis of FMO controls.

### Quantification of cytokines in culture supernatants

Cultural supernatants were harvested after 120 hours and frozen at -20°C. The cytokines IFNγ and IL10 were measured using the eBioscience ELISA ready-SET-go kit according to the manufacturer’s recommendations. The detection limits were 10 pg/ml for IL10 and 20 pg/ml for IFNγ. For statistical analysis, samples with optical density readings below the limit of the standard curve of the assay were assigned a value half that of the detection level.

### Statistics

Two groups were compared using the student’s t-test. For comparison of several parameters between two groups, t-tests with Holm-Sidak correction for multiple comparisons were used (Graph Pad Prism 7). Three or more groups were compared by ANOVA with the Bonferroni *post hoc* test. For data that was not normally distributed, the Wilcoxon matched pairs test or Friedman test with Dunn´s multiple comparison test was used. Correlation analysis was conducted by calculating Pearson’s product-moment correlation coefficient. IFNγ/IL10 cytokine combinations displayed in pie charts were compared between the two groups by performing a partial permutation test in SPICE [73].

### Study approval

Ethical approval was obtained from the Ethikkommission Hamburg. Written informed consent was obtained from all enrolled participants prior to inclusion in the study.

## Supporting Information

S1 Table Clinical details of the enrolled malaria patients. (DOCX)

S2 Table Overview of functional assays. (DOCX)

S1 Fig Foxp3 expression in malaria patients and healthy controls. Blood samples from acute malaria patients and healthy controls were analyzed *ex-vivo* for the expression of Foxp3 on CD4^+^ T cells by flow cytometry. Scatter plots show the frequency of Foxp3^+^ CD4^+^ T cells as percentage of CD4^+^ T cells for all analyzed donors (n = 12). Horizontal bars represent means. *, P = 0.025 (unpaired t-test).
(TIF)

S2 Fig CTLA4 and PD1 expression on CD8^+^ T cells. Blood samples from acute malaria patients and healthy controls were analyzed *ex-vivo* for the expression of PD1 and intracellular CTLA4 on CD8^+^ T cells by flow cytometry. Scatter plots show the frequency of CTLA4^+^ (A) and PD1^+^CD4^+^ T cells (B) as percentage of CD8^+^ T cells for all analyzed donors. Horizontal bars represent means. ***, P < 0.001; NS, P = 0.06 (t-test with Bonferroni correction for multiple comparisons).
(TIF)

S3 Fig Gating strategies for CTLA4 and PD1 on CD4^+^ T cells. A) Scatter gate to exclude debris and gate for lymphocytes, B) singlets gate to exclude doublets, C) gate on CD3 for T cells, D) gate on CD4 and CD8, E) FMO-based gate for CTLA4, F) FMO-based gate for PD1.
(TIF)

S4 Fig Expression of coinhibitory ligands on monocytes, B cells and T cells. Blood samples from acute malaria patients and healthy controls were analyzed *ex-vivo* for the expression of PDL1, PDL2, CD80 and CD86 on CD14^+^ monocytes, CD19^+^ B cells and CD 3^+^ T cells. A) The histogram shows the PDL1 expression on CD14^+^ monocytes for one representative donor. The filled histogram represents a healthy donor; the open histogram shows a malaria patient. Expression levels were compared between malaria patients and healthy volunteers. Box and whisker plots show the geometric mean fluorescence intensity of coinhibitory ligands on CD14^+^ monocytes (B), CD19^+^ B cells (C) and CD 3^+^ T cells for all analyzed donors. (n = 15) ***, P< 0,001; *, P <0.01 (t-tests with Holm-Sidak correction for multiple comparisons).
(TIF)

S5 Fig Ki67expression on CD4^+^ T cells. Blood samples from acute malaria patients and healthy controls were analyzed *ex-vivo* for the intracellular expression of Ki67 in CD4^+^ T cells by flow cytometry. Scatter plots show the frequency of Ki67 as percentage of CD4^+^ T cells or all analyzed donors. Horizontal bars represent means. **, P = 0.003 (unpaired t-test).
(TIF)

S6 Fig Proliferation in response to Pf antigens is preferentially found among the PD1^+^ and CTLA4^+^ CD4^+^ T cells. PBMC of malaria patients were labeled with CFSE and stimulated with/without iRBC (unstimulated versus iRBC). Cells were first gated for CD4^+^ T cells. Proliferating cells were further analyzed for expression of CTLA4 and PD1. The assay is shown here for two representative malaria patients out of 3. The other patient is presented in Fig 2.
(TIF)

S7 Fig Gating for intracellular cytokine staining. A) Scatter gate to exclude debris and gate for lymphocytes, B) singlets gate to exclude doublets, C) exclusion of dead cells and gating for CD3 D) gate on CD4, E) FMO-based gate for IFNγ, F) FMO-based gate for IL10.
(TIF)

S8 Fig Intracellular staining for IFNγ^+^ and IL10^+^ CD4^+^ T cells and Tbet. A) PBMC from patients with acute malaria were stimulated with iRBC and addition of Brefeldin A/Monensin for 18h. Left: The dotplot shows intracellular staining for IFNγ and IL10 in response to iRBC after gating for CD4^+^ T cells. IFNγ^+^ and IL10^+^ CD4^+^ T cells were further analyzed for expression of PD1 and Tbet. One representative patient out of three is shown. B) PBMC from patients with acute malaria were stimulated with CMV, PMA/Ionomycin or anti-CD3/28 and addition of Brefeldin A/Monensin for 18h or 12h (PMA/Ionomycin). The dotplots show intracellular staining for IFNγ and IL10 in response to these stimulans after gating for CD4^+^ T cells. One representative patient out of three is shown.
(TIF)

S9 Fig The ratio of IFNγ and IL10 production in patients with complicated and uncomplicated malaria. The ratio of IFNγ^+^ to IFNγ^+^ IL10^+^ and IL10^+^CD4^+^ T cells as detected by intracellular flow cytometry staining were analyzed in patients with uncomplicated malaria (n = 8, left) and patients with severe cerebral malaria (n = 3, right). The pie charts express the proportion of IFNγ^+^ (blue), IFNγ^+^ IL10^+^ (red) and IL10^+^ CD4^+^ T cells (green) of the net-IFNγ and IL10 cytokine response to iRBC. NS, P = 0.16 (partial permutation test).
(TIF)

S10 Fig Cell sorting and purity of sorted cells. Magnetic bead sorted CD4^+^ T cells were surface stained for CD25, CD127, PD1 and CTLA4. A) nT_regs_ were excluded based on high expression of CD25 and low expression of CD127 (CD25^+^CD127^dim^ gate). B) Remaining cells were gated and sorted for PD1^+^ cells. C) Sorted PD1^+^ CD4^+^ T cells were subsequently stained intracellularly for CTLA4. Sorted cells showed 50–95% purity for expression of PD1 and intracellular CTLA4.
(TIF)

S11 Fig Proliferation and suppression by nT_regs_, sorted PD1^-^ subsets or in response to CMV. Cell cultures were set up by stimulating CD4^+^ T cells with anti-CD3/28 (A; B and C), or CMV (D) with/without addition of FACS sorted T cells (ratio 1:0, 1:1). ^3^H-Thymidine uptake was measured after 120 hrs of culture to assess cell proliferation. Cultures were performed in triplicates unless there were insufficient cells, and values represent mean + SE. A) shows the absolute proliferation counts per minute for 2.5 x 10^4^ anti-CD3/28 stimulated CD4^+^ T cells (ratio 1:0) and for PD1^+^CTLA4^+^CD4^+^ T cells (0:1 and 1:1), compared to CD25^+^CD127^dim^ nT_regs_ (0:1 and 1:1). One representative malaria patient of three (for CD25^+^CD127^dim^ nT_regs_) is shown. B) shows the pooled results for all malaria patients, for whom proliferation and suppression by CD25^+^CD127^dim^ nT_regs_ was assessed. The net (stimulated minus spontaneous) proliferation counts of CD4^+^ T cells were set at 100%. Proliferation of other cell conditions are expressed as percentage of net proliferation counts of CD4^+^ T cells (ratio 1:0). C) shows the absolute proliferation counts per minute for 2.5 x 10^4^ anti-CD3/28 stimulated CD4^+^ T cells (ratio 1:0), 2.5 x 10^4^ anti-CD3/28 stimulated sorted PD1^-^ CD4^+^ T cells, 2.5 x 10^4^ PD1^+^CTLA4^+^CD4^+^ T cells (0:1) and 2.5 x 10^4^ CD4^+^ T cells with equal numbers (ratio 1:1) of PD1^-^CD4^+^ T cells or PD1^+^CTLA4^+^CD4^+^ T cells for one representative donor out of 3. Add-back of sorted PD1^-^CD4^+^ T cells was included as a control condition to exclude an unspecific suppressive effect caused by cell sorting. D) For antigen-specific stimulation, 10^5^ CD4^+^ T cells were stimulated with equal numbers of irradiated feeder cells and with/without equal numbers of PD1^+^CTLA4^+^CD4^+^ T cells. The diagrams show one representative suppression assay of 2, using CMV.
(TIF)

S12 Fig No difference in T cell phenotype and regulatory function between malaria patients of German and West African origin. A) PD1 and CTLA4 expression on CD4^+^ T cells were compared between malaria patients of known German or West African origin. No significant difference was determined using t-tests with Holm-Sidak correction for multiple comparisons (P = 0.8). B) The suppressive function of PD1^+^CD4^+^ T cells, presented in Fig 4B, was now differentiated between patients of German and West African origin. The net (stimulated minus spontaneous) proliferation counts for 2.5 x 10^4^ CD4^+^ T cells (CD4^+^:PD1^+^ 1:0), stimulated with anti-CD3/28, were set as 100%. Proliferation results for CD4^+^:PD1^+^ 1:1 are expressed as percentage of net proliferation counts of CD4^+^ T cells (CD4^+^:PD1^+^ 1:0).
(TIF)

S13 Fig PD1^+^CTLA4^+^CD4^+^ T cells do not show a LAG3^+^CD49b^+^ Tr1-like phenotype. PD1^+^CTLA4^+^CD4^+^ T cells CD4^+^ T cells were analyzed for the expression of CD49b and LAG3, which are markers for Tr1 regulatory T cells. Left: CD4^+^ T cells were analyzed for PD1 and CTLA4 expression. The gated PD1^+^ population was further analyzed for the expression of CD49b and LAG3 (right). One representative malaria patient of three is shown.
(TIF)
