# Supplementary material for: Acute Malaria Induces PD1+CTLA4+ Effector T Cells with Cell-Extrinsic Suppressor Function
Source: PLoS Pathog. 2016 Nov 1;12(11):e1005909. doi: 10.1371/journal.ppat.1005909 (PMC5089727; doi:10.1371/journal.ppat.1005909)
Supplement: S1 Table — (DOCX) [file ppat.1005909.s001.docx]

**S1 Table: Clinical details of the 25 enrolled malaria patients**

| Sample  number | Age | Sex | Country of origin | Country of residence | Country of exposure | Blood donation for functional assays | Parasi-temia | Hemo-globin g/l | Leukocytes  X 10^3^/µl | Thrombo-cytes  X 10^3^/µl | CRP mg/l | Severe  malaria | Treat-  ment |
| --- | --- | --- | --- | --- | --- | --- | --- | --- | --- | --- | --- | --- | --- |
| 1 | 43 | F | Ghana | Germany | Ghana | Day 4 | 1.5% | 9.8 | 3.8 | 191 | 39 | n | AP |
| 2 | 24 | M | Germany | Germany | Vanuatu | Day 2 | < 1% | 13.6 | 4.6 | 101 | 63 | n | AP |
| 3 | 22 | F | Germany | Germany | Togo | Day 3 | 1% | 13.1 | 6.4 | 134 | 20 | n | DHA-PQP |
| 4 | 34 | M | Ghana | Germany | Ghana | Day 1 | 1% | 14.2 | 6.3 | 71 | 101 | n | AP |
| 5 | 30 | F | Germany | Germany | Togo/Ghana | Day 3 | < 1% | 13.8 | 3.1 | 49 | 82 | n | DHA-PQP |
| 6 | 26 | F | Germany | Germany | Burkina Faso | Day 2 | < 1% | 10.5 | 2.8 | 62 | 40 | n | DHA-PQP |
| 7 | 40 | F | Ghana | Germany | Ghana | Day 4 | < 0.5% | 11.7 | 5.8 | 40 | 231 | n | AP |
| 8 | 41 | M | Cote d´Ivoire | Germany | Cote d´Ivoire | Day 1 | < 1% | 12 | 3.8 | 91 | 109 | n | AP |
| 9 | 33 | M | Gambia | Germany | Gambia | Day 3 | <1% | 10.5 | 4.5 | 193 | 127 | n | AP |
| 10 | 64 | M | Germany | Nigeria | Nigeria | Day 4 | 1.5% | 17.8 | 5.0 | 42 | 143 | y (CM) | AS+AP |
| 11 | 49 | M | Nigeria | Germany | Nigeria | Day 2 | < 1% | 13.9 | 4.6 | 102 | 33 | n | AP |
| 12 | 48 | M | Nigeria | Germany | Nigeria | Day 1 | < 1% | 14 | 2.2 | 13 | 268 | n | DHA-PQP |
| 13 | 50 | M | Ghana | Germany | Ghana | Day 3 | 4.5% | 13.1 | 3.4 | 26 | 246 | n | DHA-PQP |
| 14 | 39 | M | Cameroon | Germany | Cameroon | Day 3 | < 1% | 14 | 2.8 | 96 | 101 | y (CM) | AS + AP |
| 15 | 55 | F | Ghana | Germany | Ghana | Day 2 | <1% | 11.7 | 4.3 | 210 | 28 | n | DHA-PQP |
| 16 | 48 | F | Germany | Germany | Uganda | Day 6 | 20% | 14 | 2.8 | 16 | 150 | y (HP) | AS + AP |
| 17 | 43 | M | Germany | Germany | Uganda | Day 2 | 27% | 14.6 | 4.8 | 172 | 26 | y (HP) | AS + AP |
| 18 | 70 | F | Germany | Cameroon | Cameroon | Day 6 | < 1% | 15 | 7.2 | 31 | 103 | y (CM) | AS + AP |
| 19 | 46 | M | Cote d´Ivoire | Germany | Cote d´Ivoire | Day 1 | < 1% | 12.7 | 4.6 | 58 | 60 | n | DHA-PQP |
| 20 | 38 | M | Nigeria | Germany | Nigeria | Day 3 | < 1% | 13.3 | 2.9 | 39 | 225 | n | AP |
| 21 | 52 | M | Gambia | Gambia | Gambia | Day 2 | < 1% | 14.5 | 9.6 | 116 | 108 | n | AP |
| 22 | 39 | F | Cameroon | Germany | Cameroon | Day 2 | 5% | 10.8 | 4.5 | 64 | 106 | n | AP |
| 23 | 18 | F | Germany | Germany | Togo | Day 4 | < 1% | 14.7 | 6.4 | 183 | 162 | n | DHA-PQP |
| 24 | 52 | M | Guinea | Germany | Guinea | Day 3 | 8% | 13 | 2.4 | 101 | 55 | n | AP |
| 25 | 55 | M | Germany | Germany | Ghana | Day 2 | < 1% | 14.7 | 4.8 | 43 | 227 | n | DHA-PQP |

CRP = C-reactive protein, CM = cerebral malaria, HP = hyperparasitemia

AP = atovaquone-proguanil, DHA-PQP = dihydroartemisinin-piperaquine, AS = artesunate
