# Supplementary material for: Acute Malaria Induces PD1+CTLA4+ Effector T Cells with Cell-Extrinsic Suppressor Function
Source: PLoS Pathog. 2016 Nov 1;12(11):e1005909. doi: 10.1371/journal.ppat.1005909 (PMC5089727; doi:10.1371/journal.ppat.1005909)
Supplement: S2 Table — (DOCX) [file ppat.1005909.s002.docx]

**S2 Table: Overview over functional assays**

| Sample  number | Functional assays | | | | | | | |
| --- | --- | --- | --- | --- | --- | --- | --- | --- |
|  | *Pf*- specific proliferation:  CFSE assay  (n=6) | *Pf*-specific proliferation:  ^3^H assay with/without anti-CTLA4/PDL1  (n=11) | Cytokine responses to *Pf:*  Intracellular flow cytometry analysis  (n= 17) | IFNγ response to *Pf* with/without anti- CTLA4/PDL1:  ELISA assay  (n=9) | Suppression assay: anti-CD3/28  (n=7) | Suppression assay:  iRBC  (n=6) | Suppression assay: transwell setup  (n=3) | Suppression assay:  anti-CTLA4/PDL1/IL10/ TGFβ  (n= 4) |
| 1 |  | positive | positive | positive | positive |  |  |  |
| 2 |  |  | positive |  | positive |  | positive | positive |
| 3 | neg. |  | positive |  | positive |  | positive |  |
| 4 | neg. |  | positive |  |  |  |  |  |
| 5 |  |  | positive |  |  |  |  |  |
| 6 |  |  | neg. |  |  | positive |  |  |
| 7 | positive |  | positive |  |  |  |  |  |
| 8 |  |  | neg. |  | positive |  | positive | positive |
| 9 | neg. |  | positive |  | positive | neg |  |  |
| 10 | positive |  | positive |  |  |  |  |  |
| 11 |  |  | neg. |  |  | positive |  |  |
| 12 |  | neg. | neg. |  |  |  |  |  |
| 13 |  |  | positive |  |  |  |  |  |
| 14 |  | neg. | positive | positive |  |  |  |  |
| 15 | positive | positive | neg. | positive |  |  |  |  |
| 16 |  | neg. | neg. |  |  |  |  |  |
| 17 |  | neg. |  | positive |  |  |  |  |
| 18 |  | positive | positive | positive |  |  |  |  |
| 19 |  |  |  |  |  | neg |  |  |
| 20 |  |  |  |  |  | neg |  |  |
| 21 |  | positive |  | positive |  |  |  |  |
| 22 |  | neg. |  | positive |  |  |  |  |
| 23 |  | neg. |  | positive |  | neg |  |  |
| 24 |  |  |  |  | positive |  |  | positive |
| 25 |  | neg. |  | positive | positive |  |  | positive |

neg: assay was set up, but no response (proliferation or cytokine production) to *Pf* antigens was detected in this specific assay

positive: *Pf*-specific response (proliferation or cytokine production) or anti-CD3/28 induced proliferation was detected
