# Supplementary figures and images for: Acute Malaria Induces PD1+CTLA4+ Effector T Cells with Cell-Extrinsic Suppressor Function
Source: PLoS Pathog. 2016 Nov 1;12(11):e1005909. doi: 10.1371/journal.ppat.1005909 (PMC5089727; doi:10.1371/journal.ppat.1005909)

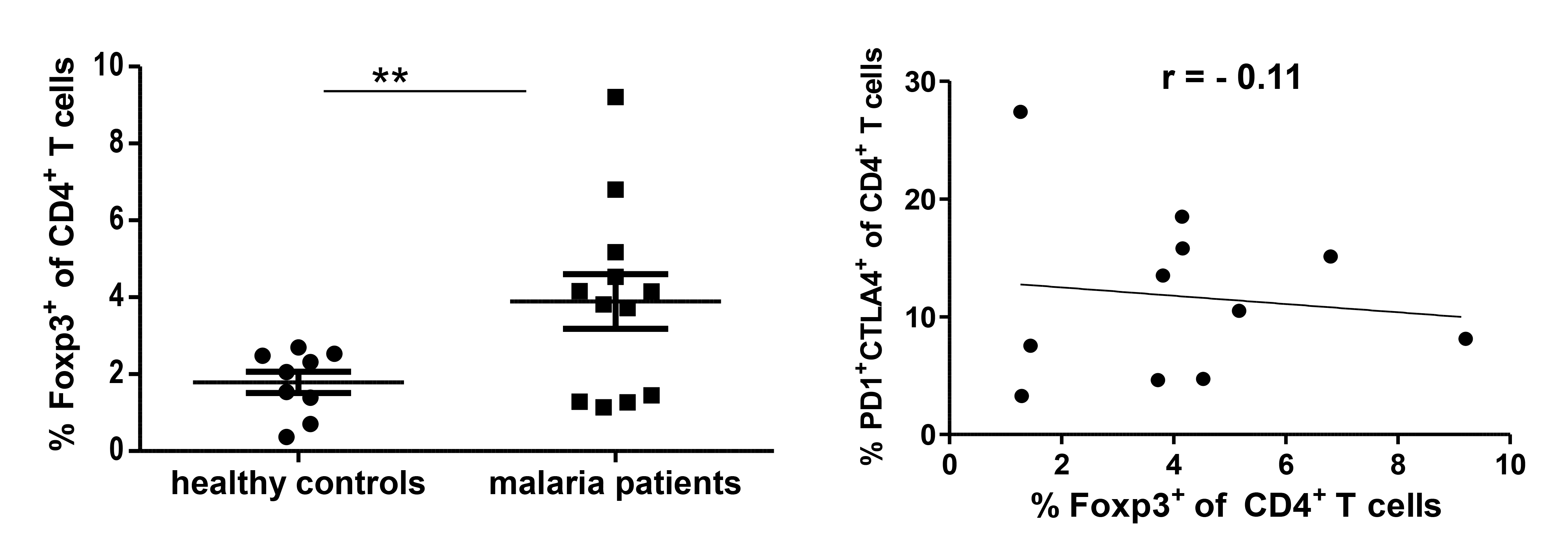

Supplement: S1 Fig — Blood samples from acute malaria patients and healthy controls were analyzed ex-vivo for the expression of Foxp3 on CD4+ T cells by flow cytometry. Scatter plots show the frequency of Foxp3+ CD4+ T cells as percentage of CD4+ T cells for all analyzed donors (n = 12). Horizontal bars represent means. *, P = 0.025 (unpaired t-test). (TIF) [file ppat.1005909.s003.tif]

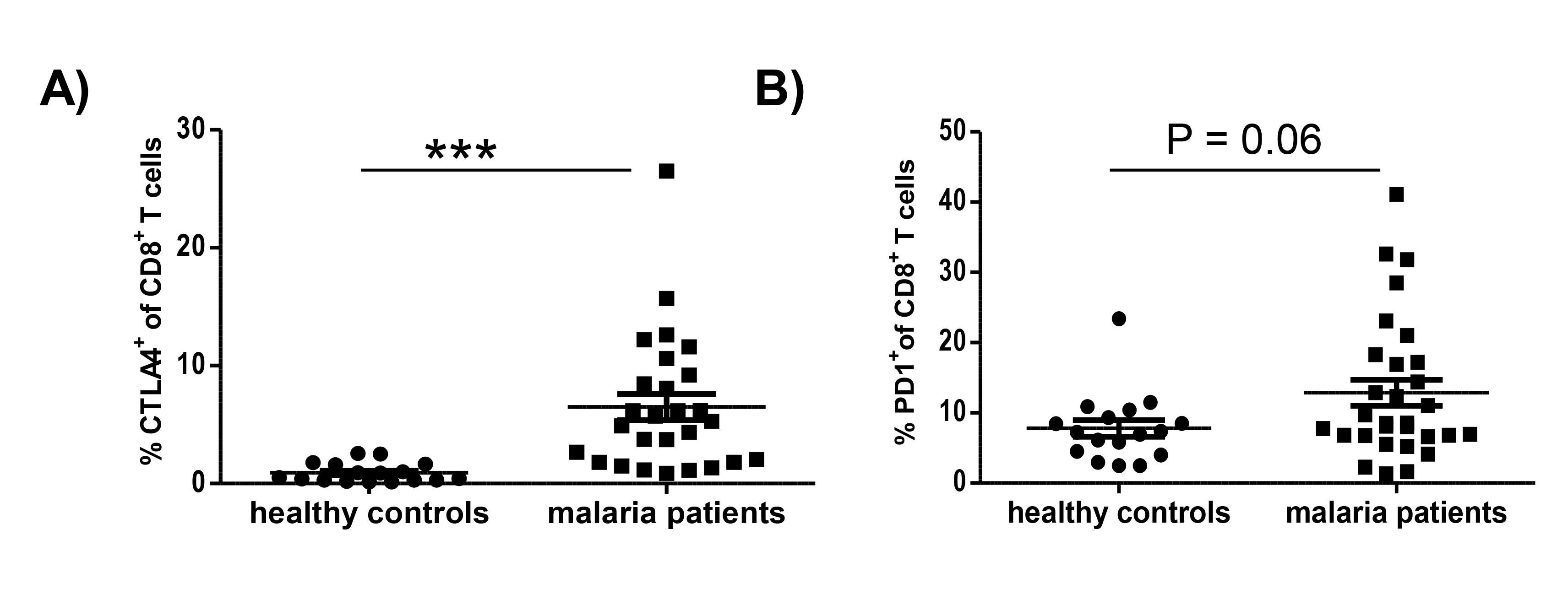

Supplement: S2 Fig — Blood samples from acute malaria patients and healthy controls were analyzed ex-vivo for the expression of PD1 and intracellular CTLA4 on CD8+ T cells by flow cytometry. Scatter plots show the frequency of CTLA4+ (A) and PD1+CD4+ T cells (B) as percentage of CD8+ T cells for all analyzed donors. Horizontal bars represent means. ***, P < 0.001; NS, P = 0.06 (t-test with Bonferroni correction for multiple comparisons). (TIF) [file ppat.1005909.s004.tif]

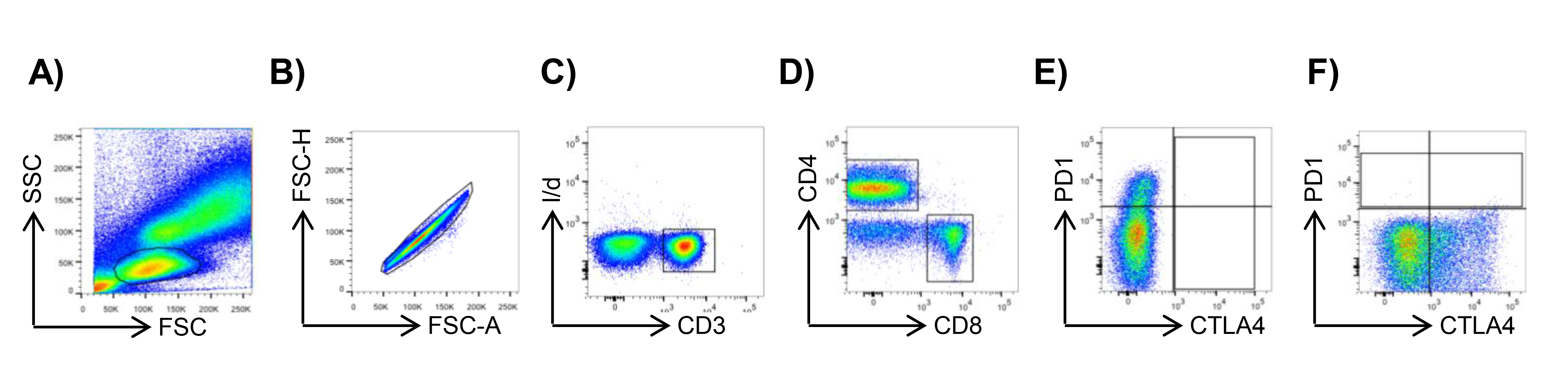

Supplement: S3 Fig — A) Scatter gate to exclude debris and gate for lymphocytes, B) singlets gate to exclude doublets, C) gate on CD3 for T cells, D) gate on CD4 and CD8, E) FMO-based gate for CTLA4, F) FMO-based gate for PD1. (TIF) [file ppat.1005909.s005.tif]

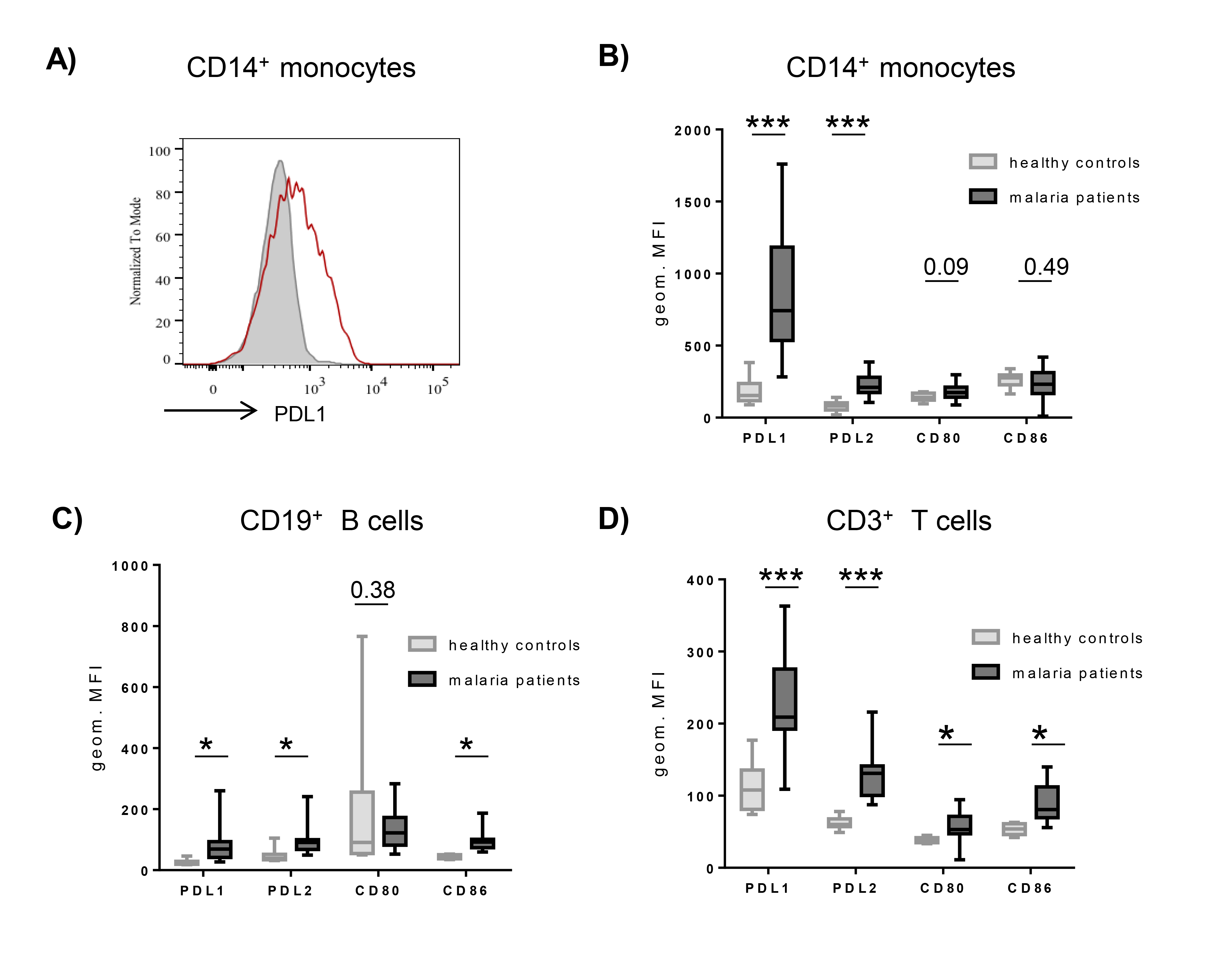

Supplement: S4 Fig — Blood samples from acute malaria patients and healthy controls were analyzed ex-vivo for the expression of PDL1, PDL2, CD80 and CD86 on CD14+ monocytes, CD19+ B cells and CD 3+ T cells. A) The histogram shows the PDL1 expression on CD14+ monocytes for one representative donor. The filled histogram represents a healthy donor; the open histogram shows a malaria patient. Expression levels were compared between malaria patients and healthy volunteers. Box and whisker plots show the geometric mean fluorescence intensity of coinhibitory ligands on CD14+ monocytes (B), CD19+ B cells (C) and CD 3+ T cells for all analyzed donors. (n = 15) ***, P< 0,001; *, P <0.01 (t-tests with Holm-Sidak correction for multiple comparisons). (TIF) [file ppat.1005909.s006.tif]

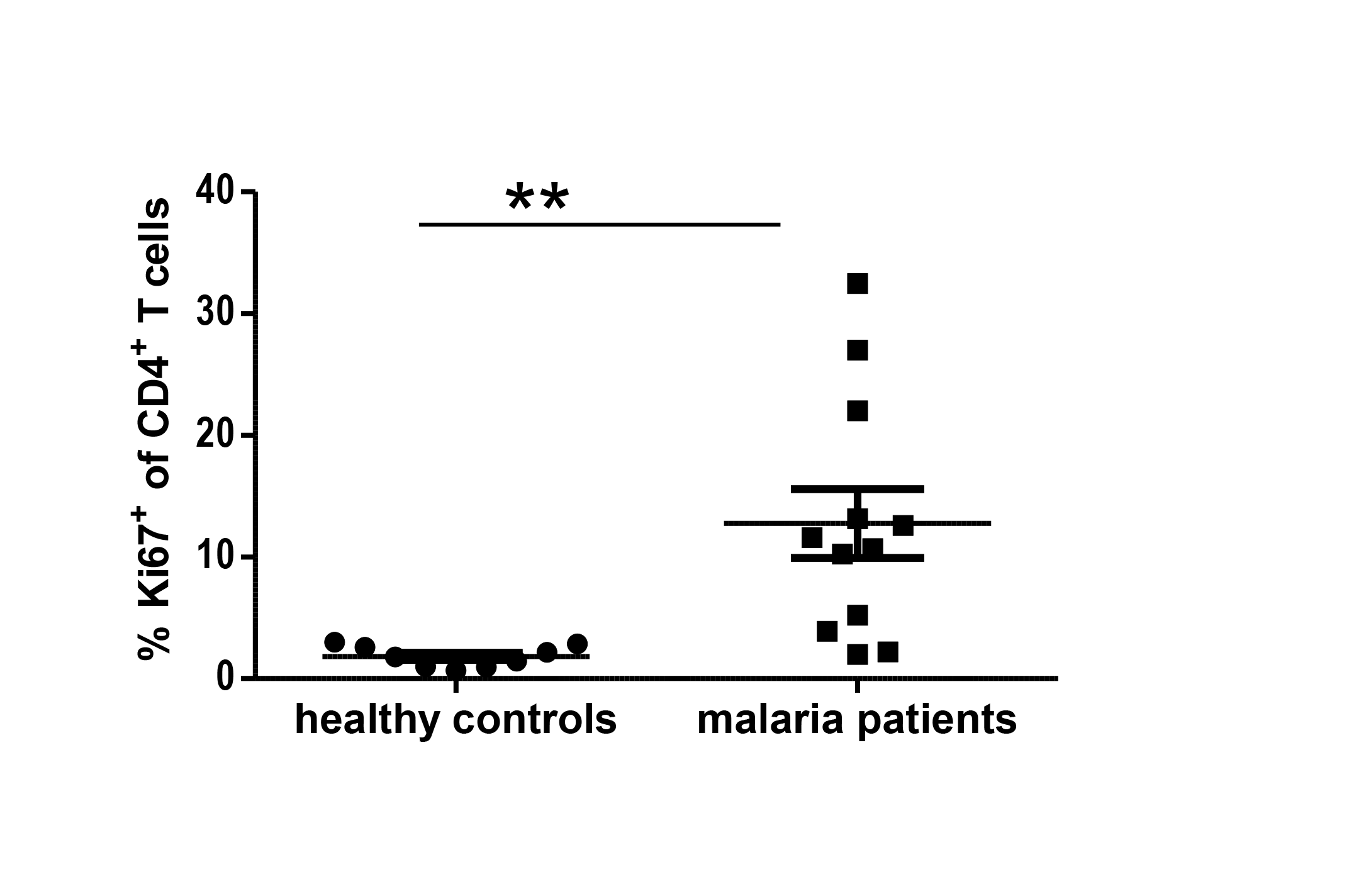

Supplement: S5 Fig — Blood samples from acute malaria patients and healthy controls were analyzed ex-vivo for the intracellular expression of Ki67 in CD4+ T cells by flow cytometry. Scatter plots show the frequency of Ki67 as percentage of CD4+ T cells or all analyzed donors. Horizontal bars represent means. **, P = 0.003 (unpaired t-test). (TIF) [file ppat.1005909.s007.tif]

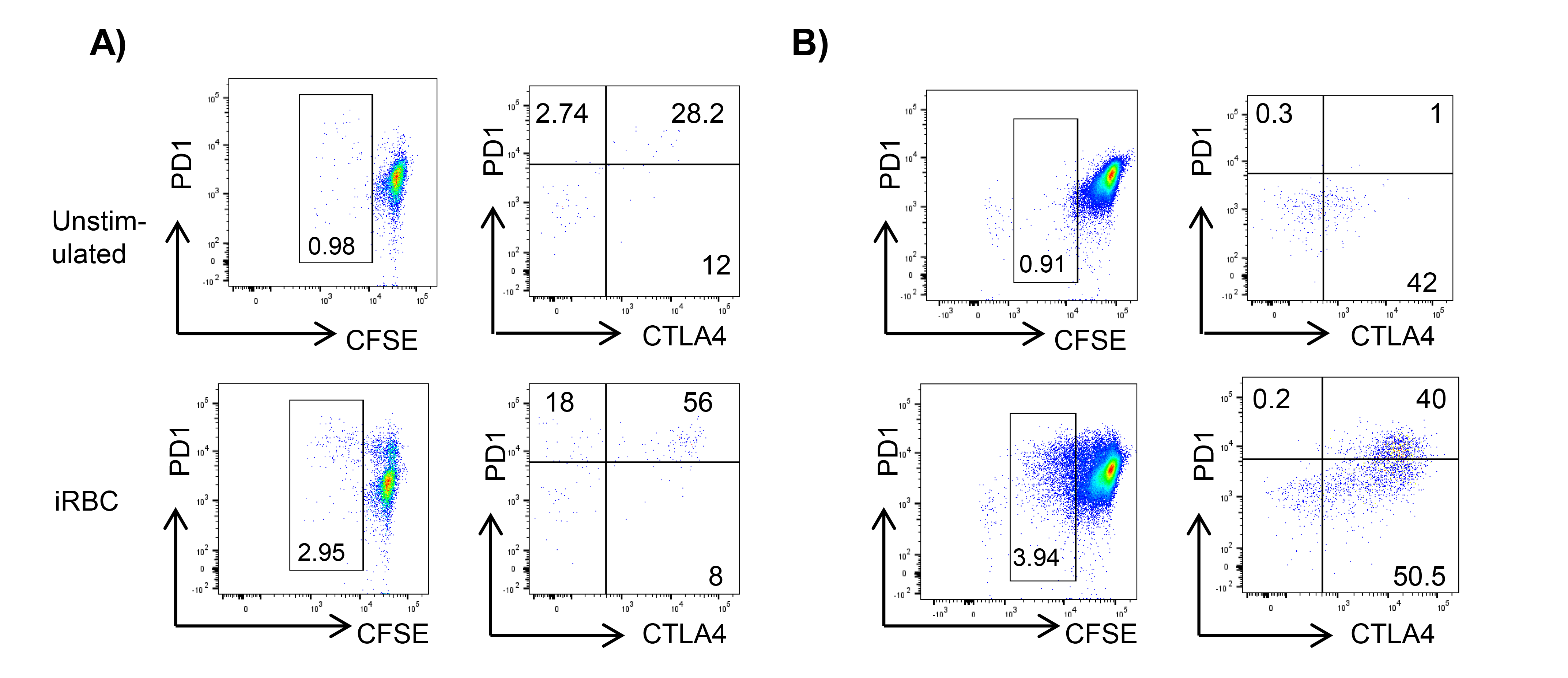

Supplement: S6 Fig — PBMC of malaria patients were labeled with CFSE and stimulated with/without iRBC (unstimulated versus iRBC). Cells were first gated for CD4+ T cells. Proliferating cells were further analyzed for expression of CTLA4 and PD1. The assay is shown here for two representative malaria patients out of 3. The other patient is presented in Fig 2. (TIF) [file ppat.1005909.s008.tif]

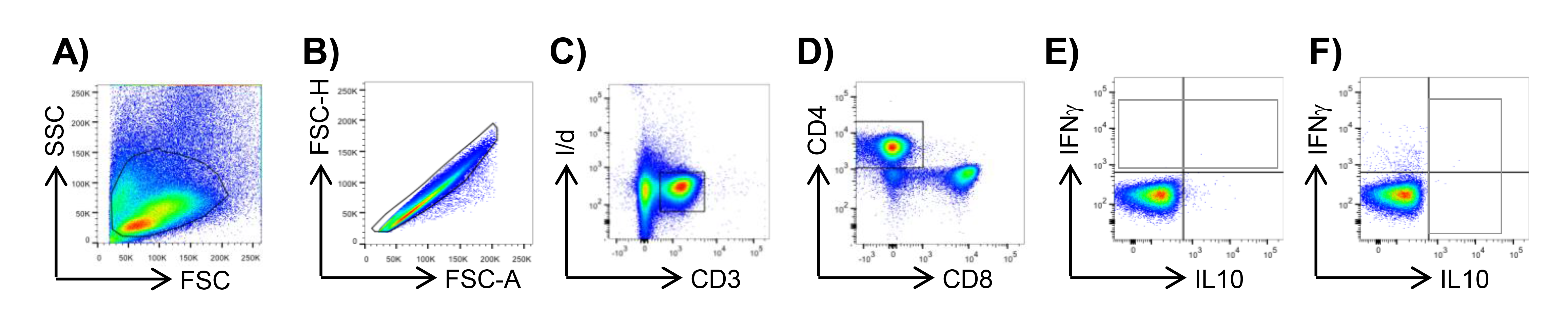

Supplement: S7 Fig — A) Scatter gate to exclude debris and gate for lymphocytes, B) singlets gate to exclude doublets, C) exclusion of dead cells and gating for CD3 D) gate on CD4, E) FMO-based gate for IFNγ, F) FMO-based gate for IL10. (TIF) [file ppat.1005909.s009.tif]

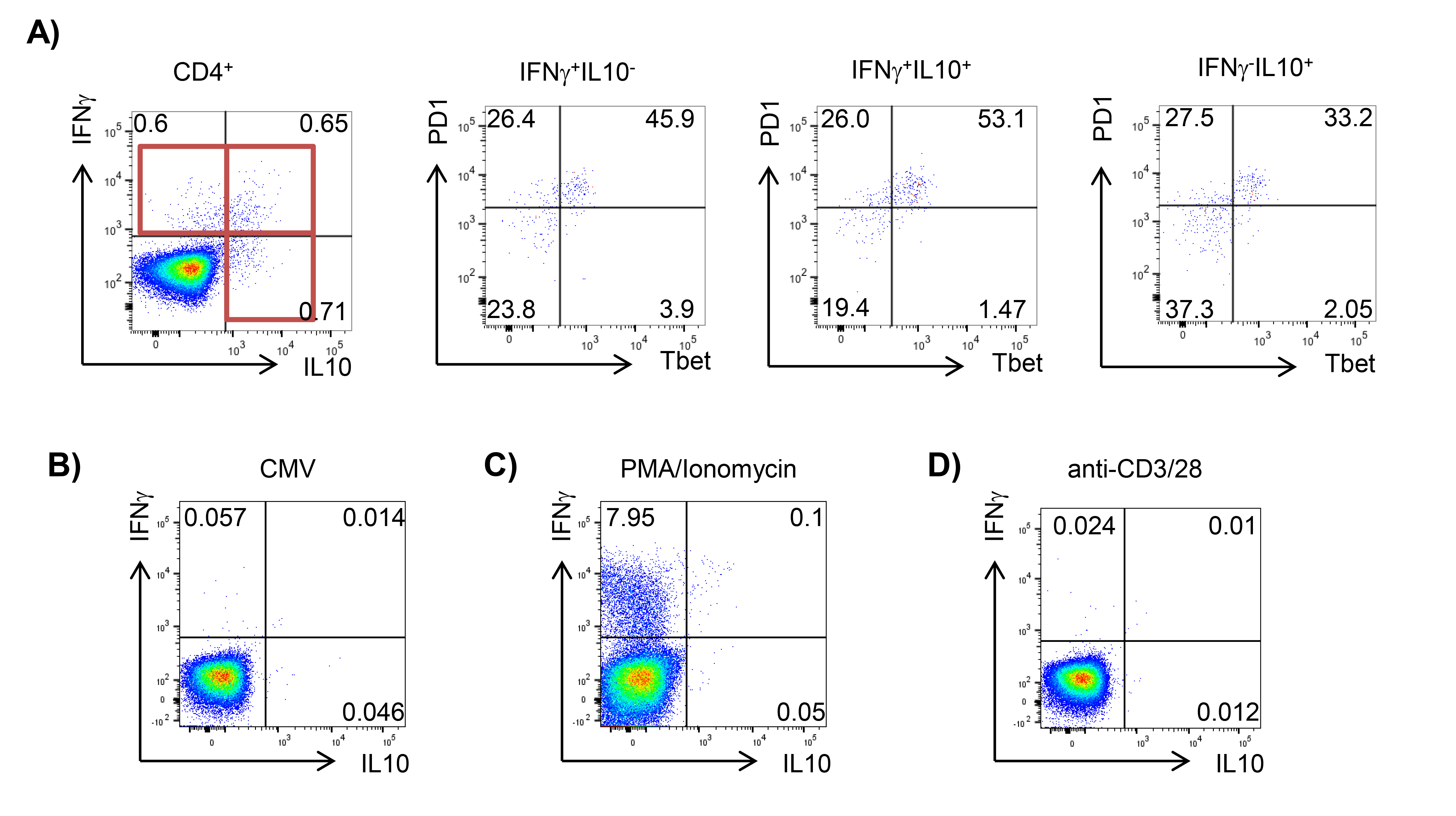

Supplement: S8 Fig — A) PBMC from patients with acute malaria were stimulated with iRBC and addition of Brefeldin A/Monensin for 18h. Left: The dotplot shows intracellular staining for IFNγ and IL10 in response to iRBC after gating for CD4+ T cells. IFNγ+ and IL10+ CD4+ T cells were further analyzed for expression of PD1 and Tbet. One representative patient out of three is shown. B) PBMC from patients with acute malaria were stimulated with CMV, PMA/Ionomycin or anti-CD3/28 and addition of Brefeldin A/Monensin for 18h or 12h (PMA/Ionomycin). The dotplots show intracellular staining for IFNγ and IL10 in response to these stimulans after gating for CD4+ T cells. One representative patient out of three is shown. (TIF) [file ppat.1005909.s010.tif]

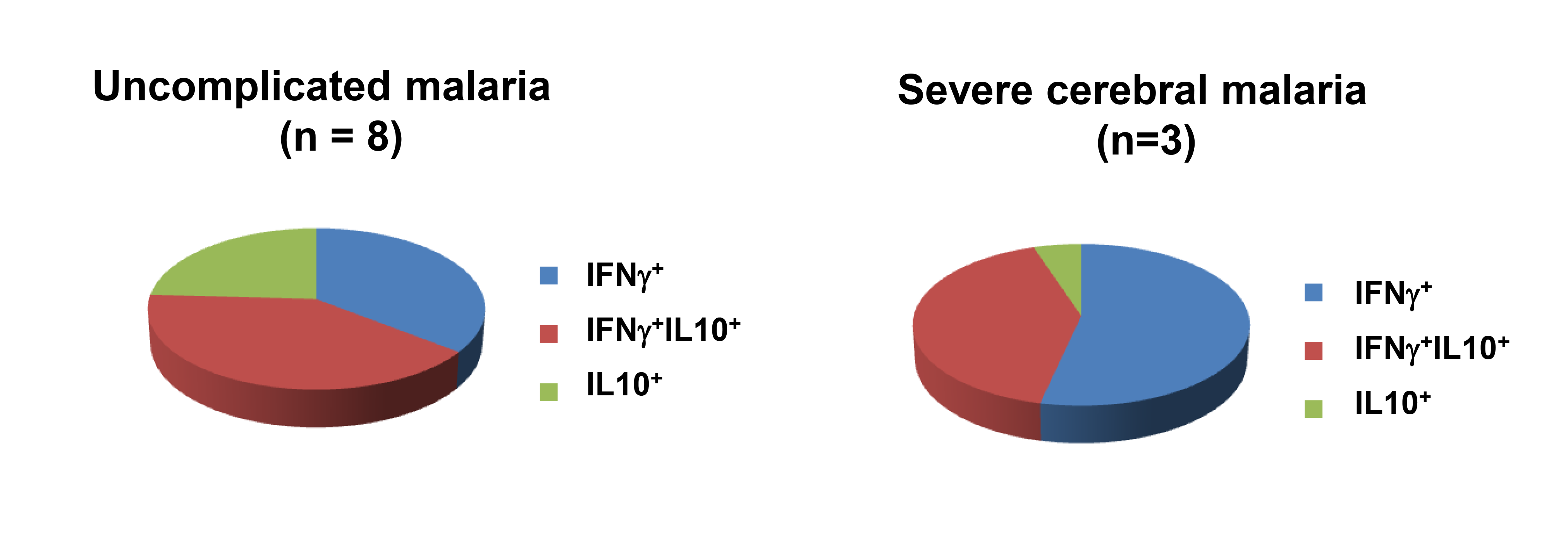

Supplement: S9 Fig — The ratio of IFNγ+ to IFNγ+ IL10+ and IL10+CD4+ T cells as detected by intracellular flow cytometry staining were analyzed in patients with uncomplicated malaria (n = 8, left) and patients with severe cerebral malaria (n = 3, right). The pie charts express the proportion of IFNγ+ (blue), IFNγ+ IL10+ (red) and IL10+ CD4+ T cells (green) of the net-IFNγ and IL10 cytokine response to iRBC. NS, P = 0.16 (partial permutation test). (TIF) [file ppat.1005909.s011.tif]

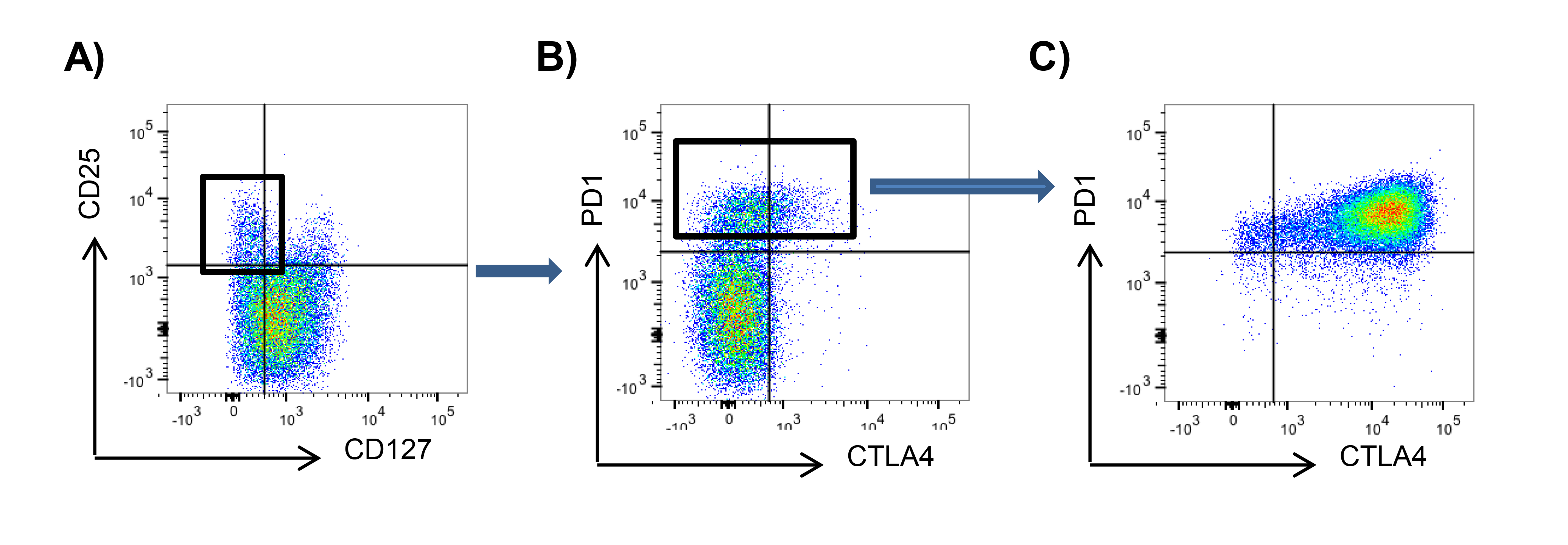

Supplement: S10 Fig — Magnetic bead sorted CD4+ T cells were surface stained for CD25, CD127, PD1 and CTLA4. A) nTregs were excluded based on high expression of CD25 and low expression of CD127 (CD25+CD127dim gate). B) Remaining cells were gated and sorted for PD1+ cells. C) Sorted PD1+ CD4+ T cells were subsequently stained intracellularly for CTLA4. Sorted cells showed 50–95% purity for expression of PD1 and intracellular CTLA4. (TIF) [file ppat.1005909.s012.tif]

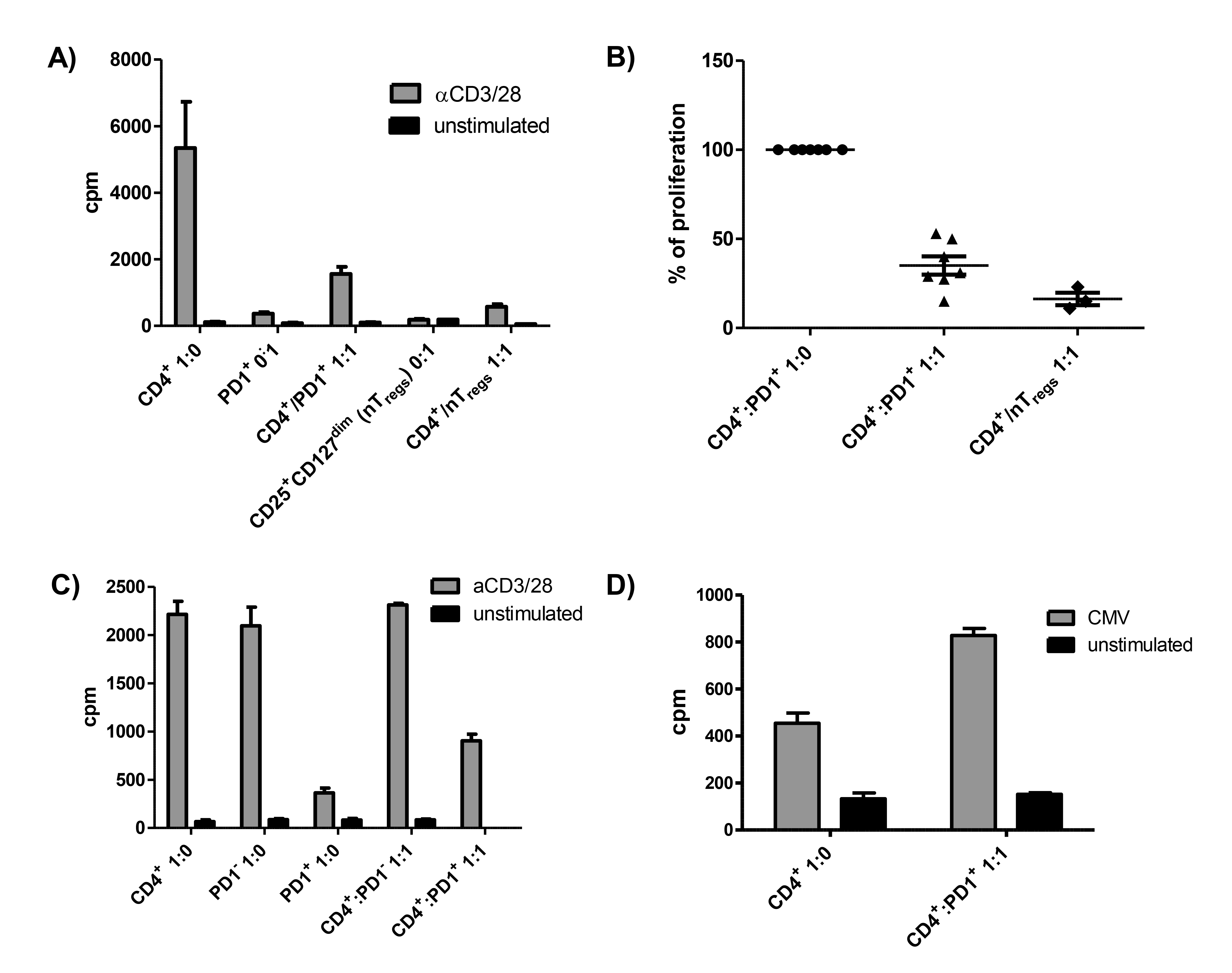

Supplement: S11 Fig — Cell cultures were set up by stimulating CD4+ T cells with anti-CD3/28 (A; B and C), or CMV (D) with/without addition of FACS sorted T cells (ratio 1:0, 1:1). 3H-Thymidine uptake was measured after 120 hrs of culture to assess cell proliferation. Cultures were performed in triplicates unless there were insufficient cells, and values represent mean + SE. A) shows the absolute proliferation counts per minute for 2.5 x 10^4^ anti-CD3/28 stimulated CD4+ T cells (ratio 1:0) and for PD1+CTLA4+CD4+ T cells (0:1 and 1:1), compared to CD25+CD127dim nTregs (0:1 and 1:1). One representative malaria patient of three (for CD25+CD127dim nTregs) is shown. B) shows the pooled results for all malaria patients, for whom proliferation and suppression by CD25+CD127dim nTregs was assessed. The net (stimulated minus spontaneous) proliferation counts of CD4+ T cells were set at 100%. Proliferation of other cell conditions are expressed as percentage of net proliferation counts of CD4+ T cells (ratio 1:0). C) shows the absolute proliferation counts per minute for 2.5 x 10^4^ anti-CD3/28 stimulated CD4+ T cells (ratio 1:0), 2.5 x 10^4^ anti-CD3/28 stimulated sorted PD1- CD4+ T cells, 2.5 x 10^4^ PD1+CTLA4+CD4+ T cells (0:1) and 2.5 x 10^4^ CD4+ T cells with equal numbers (ratio 1:1) of PD1-CD4+ T cells or PD1+CTLA4+CD4+ T cells for one representative donor out of 3. Add-back of sorted PD1-CD4+ T cells was included as a control condition to exclude an unspecific suppressive effect caused by cell sorting. D) For antigen-specific stimulation, 105 CD4+ T cells were stimulated with equal numbers of irradiated feeder cells and with/without equal numbers of PD1+CTLA4+CD4+ T cells. The diagrams show one representative suppression assay of 2, using CMV. (TIF) [file ppat.1005909.s013.tif]

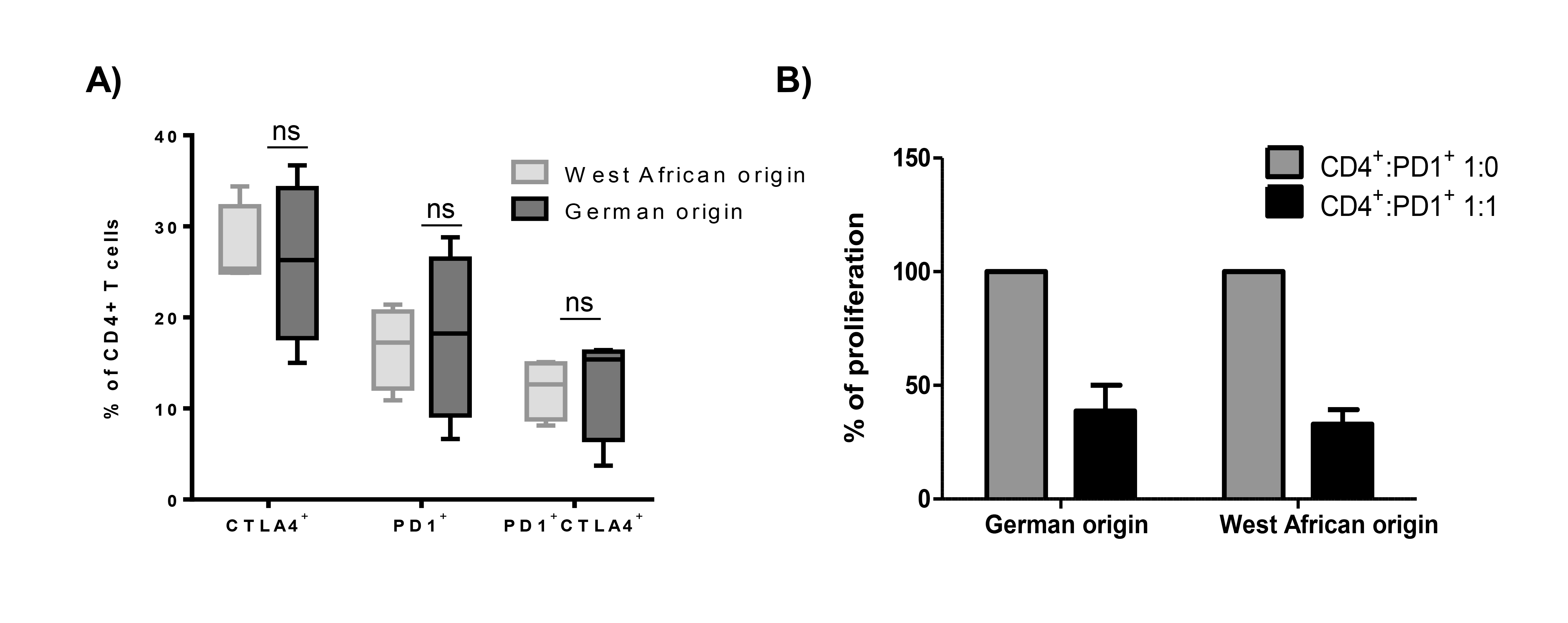

Supplement: S12 Fig — A) PD1 and CTLA4 expression on CD4+ T cells were compared between malaria patients of known German or West African origin. No significant difference was determined using t-tests with Holm-Sidak correction for multiple comparisons (P = 0.8). B) The suppressive function of PD1+CD4+ T cells, presented in Fig 4B, was now differentiated between patients of German and West African origin. The net (stimulated minus spontaneous) proliferation counts for 2.5 x 10^4^ CD4+ T cells (CD4+:PD1+ 1:0), stimulated with anti-CD3/28, were set as 100%. Proliferation results for CD4+:PD1+ 1:1 are expressed as percentage of net proliferation counts of CD4+ T cells (CD4+:PD1+ 1:0). (TIF) [file ppat.1005909.s014.tif]

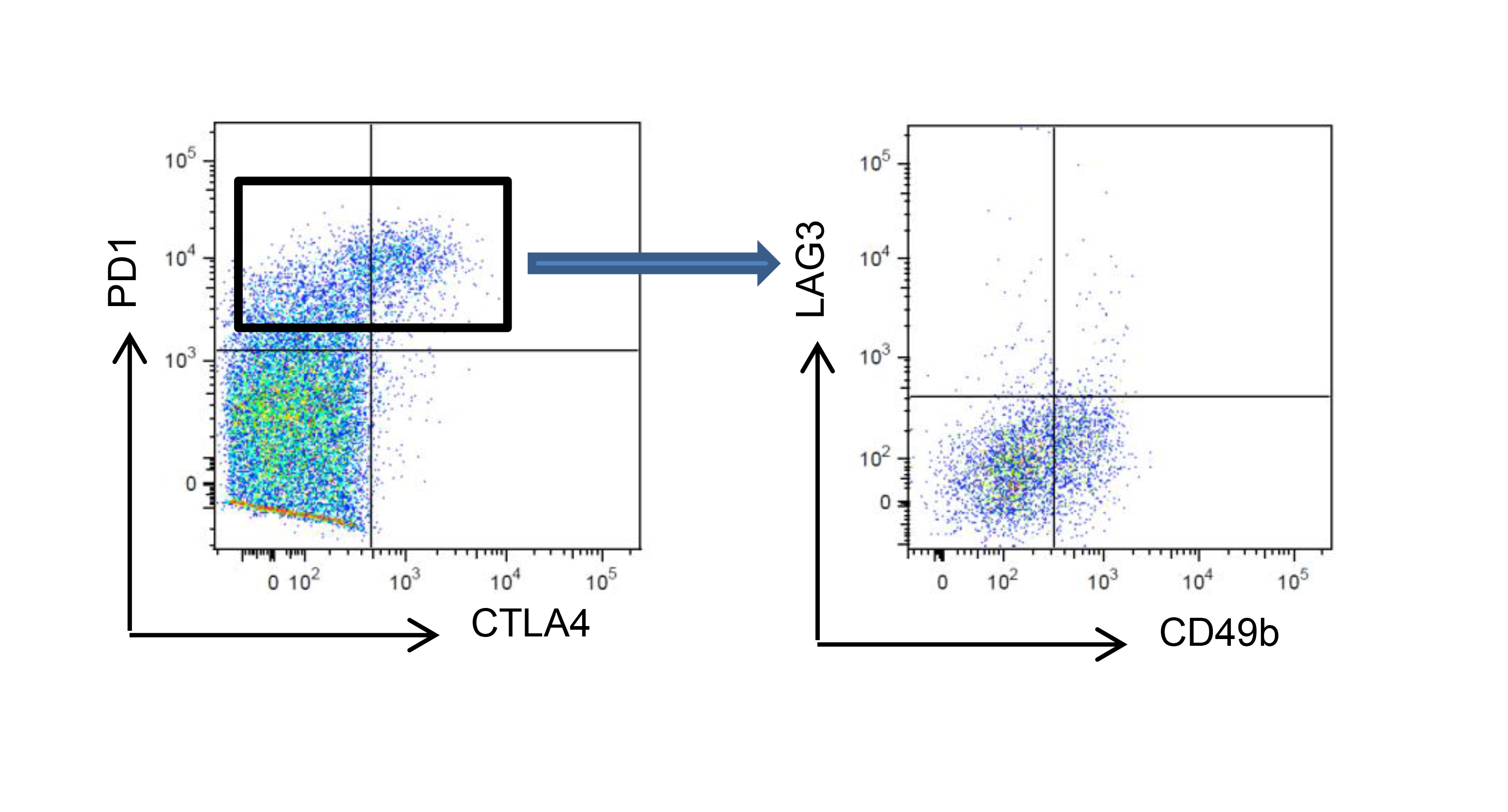

Supplement: S13 Fig — PD1+CTLA4+CD4+ T cells CD4+ T cells were analyzed for the expression of CD49b and LAG3, which are markers for Tr1 regulatory T cells. Left: CD4+ T cells were analyzed for PD1 and CTLA4 expression. The gated PD1+ population was further analyzed for the expression of CD49b and LAG3 (right). One representative malaria patient of three is shown. (TIF) [file ppat.1005909.s015.tif]
